# Synthesis of Pyrido[2,3‐d]Azolopyrimidinones: Design and Epidermal Growth Factor Receptor‐Targeted Molecular Docking Toward Novel Anticancer Leads

**DOI:** 10.1002/open.202500555

**Published:** 2025-11-23

**Authors:** Sobhi M. Gomha, Sami A. Al‐Hussain, Basant Farag, AbdElAziz A. Nayl, Wesam Hussein, Abdelwahed R. Sayed, Magdi E. A. Zaki

**Affiliations:** ^1^ Department of Chemistry Faculty of Science Islamic University of Madinah Madinah Saudi Arabia; ^2^ Department of Chemistry Faculty of Science Imam Mohammad Ibn Saud Islamic University (IMSIU) Riyadh Saudi Arabia; ^3^ Department of Chemistry Faculty of Science Zagazig University Zagazig Egypt; ^4^ Department of Chemistry College of Science Jouf University Sakaka Saudi Arabia; ^5^ Department of Chemistry Faculty of Science University of Beni Suef Beni Suef Egypt; ^6^ Egypt‐Japan University of Science and Technology Alexandria Egypt

**Keywords:** ADMET, anticancer agents, EGFR inhibitors, hydrazonoyl halides, molecular docking, pyridopyrimidines

## Abstract

A new class of pyrido[2,3‐d][1,2,4]triazolo[4,3‐a]pyrimidinones and pyrido[2,3‐d]thiazolo[3,2‐a]pyrimidinones was synthesized by reacting 5‐phenyl‐2‐thioxo‐2,3‐dihydropyrido[2,3‐d]pyrimidin‐4(1H)‐one with hydrazonoyl halides and α‐bromoketones via a Knoevenagel–cyclocondensation followed by heteroannulation. Structures were confirmed by elemental analysis and IR, ^1^H NMR, and MS spectroscopy. Cytotoxicity against HepG2 cells (MTT assay) revealed submicromolar activity for the most active analogs (IC_50_ 0.72–0.95 *µ*M), comparable to doxorubicin (0.65 *µ*M). Structure–activity trends indicate that ester functionalities, coumarin incorporation, and electron‐donating aryl substituents enhance potency. Molecular docking to the EGFR kinase domain showed strong predicted binding for the top analogs (scores −9.6 to −10.2 kcal mol^−1^ vs −8.7 kcal mol^−1^ for doxorubicin), highlighting key hydrogen‐bond and hydrophobic contacts with Lys745, Asp837, Arg841, and Asp855. Docking results align with the in vitro data. In silico ADMET predictions suggest favorable drug‐likeness, oral absorption, and non‐mutagenic character. These findings position the reported pyridopyrimidine scaffolds as promising EGFR‐targeted anticancer leads.

## Graphical abstract

New pyrido[2,3‐d]azolopyrimidinones were synthesized and evaluated as potent EGFR‐targeted anticancer leads. Molecular docking and cytotoxicity studies revealed strong receptor binding and submicromolar activity, highlighting this scaffold as a promising framework for future targeted drug development.

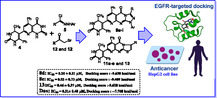

## 1 Introduction

Cancer is one of the most life‐threatening diseases worldwide, mainly because cancer cells can grow uncontrollably and spread to other organs, a process known as metastasis [1, 2]. This ability to disseminate is the main reason for the high mortality associated with the disease. Genetic mutations and disturbances in normal cellular differentiation are key drivers of tumor initiation, and various environmental and lifestyle factors—including drugs, viral infections, radiation, tobacco use, and diet—can act as triggers [3, 4]. According to the World Health Organization (WHO), cancer ranks as the second leading cause of global mortality, accounting for 9.6 million deaths in 2018. Alarmingly, this number is predicted to rise to about 13 million deaths by 2030 [5, 6].

Cancer therapies still lack selectivity, damaging healthy tissues and underscoring the need for safer, more effective agents [7]. Protein kinases are prime targets in oncology given their roles in proliferation and survival [8, 9, 10, 11, 12, 13, 14, 15], with EGFR a central focus in multiple tumors [16, 17, 18, 19]. Resistance, notably T790M, drives sequential EGFR inhibitor development from first‐ to later‐generation agents [20, 21, 22, 23, 24, 25, 26, 27, 28, 29].

In medicinal chemistry, heterocyclic systems are considered privileged scaffolds because of their structural flexibility and wide spectrum of biological activities [30, 31, 32, 33]. Among them, pyridopyrimidines stand out for their diverse pharmacological properties, including anticancer, antiviral, and central nervous system activities [34, 35, 36]. Examples such as Dilmapimod, investigated for rheumatoid arthritis [37], and Palbociclib, an FDA‐approved breast cancer drug [38, 39], demonstrate the therapeutic potential of this class. Notably, pyrido[2,3‐d]pyrimidines are capable of binding to the ATP pocket of EGFR and effectively inhibiting its activity [40]. Some derivatives have shown potent EGFR inhibition with IC_50_ values in the submicromolar or even nanomolar range, in some cases outperforming standard inhibitors like erlotinib and gefitinib [41, 42].

Structural studies indicate that most EGFR inhibitors share a characteristic Y‐shaped architecture [43], with pharmacophoric elements distributed across the adenine pocket and adjacent hydrophobic regions of the ATP‐binding site [44, 45]. Typically, a flat heteroaromatic system anchors in the adenine pocket through hydrogen bonds with key residues such as Met769, Thr790, and Thr854 [46], while hydrophobic substituents extend into regions I and II to maximize binding affinity [47, 48].

Dysregulated EGFR (ErbB) signaling drives many cancers, and several small‐molecule EGFR inhibitors are clinically approved. We therefore used molecular docking to predict target binding and contextualize our in vitro antitumor results [48]. The most active derivatives showed EGFR‐consistent interactions and favorable in silico ADMET profiles, indicating drug‐like potential.

**FIGURE 1:**
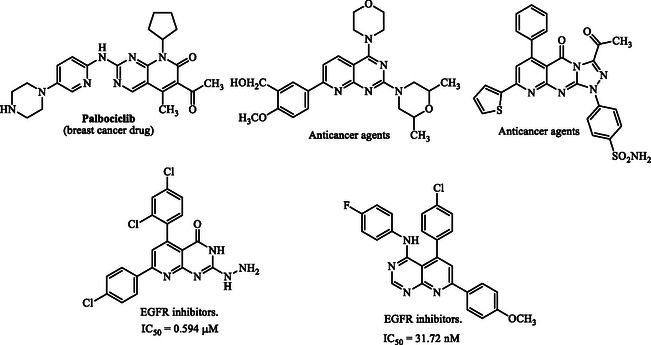
Representative biologically active pyridopyrimidine derivatives as anticancer agents.

Building on prior work in heterocyclic anticancer agents [49, 50, 51, 52, 53, 54, 55, 56, 57], we designed and synthesized pyrido[2,3‐d]azolopyrimidinones and evaluated their cytotoxicity against HepG2 by MTT (doxorubicin as a cytotoxic control). Given the clinical relevance of EGFR, we performed docking to rationalize kinase‐domain interactions. The fused azole–pyridopyrimidinone scaffold affords a rigid tricyclic framework expected to enhance binding and guide SAR. This study reports the synthesis, biological evaluation, and EGFR‐focused docking of these candidates as potential anticancer leads Figure 1.

## 2 Results and Discussion

**SCHEME 1:**
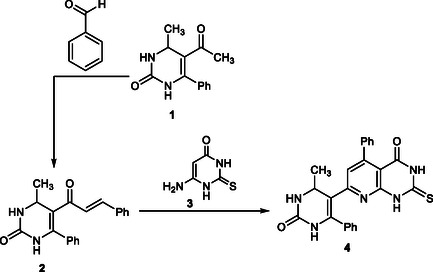
Synthesis of 5‐phenyl‐2‐thioxo‐2,3‐dihydropyrido[2,3‐d]pyrimidinone derivative **4**.

**SCHEME 2:**
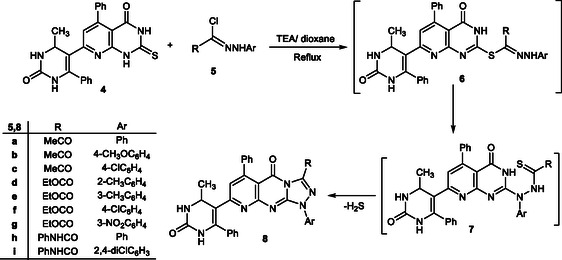
Synthesis of triazolopyridopyrimidines **8a–i**.

**SCHEME 3:**
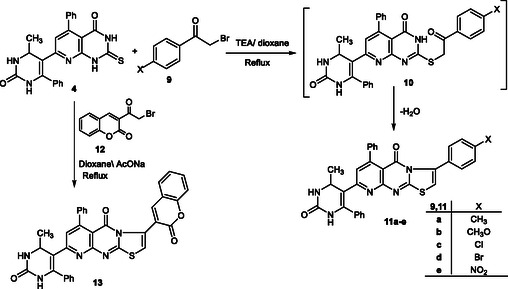
Synthesis of thiazolopyridopyrimidines **11a–e** and **13**.

In this study, a series of fused pyridopyrimidine scaffolds were efficiently synthesized through a versatile strategy starting from thione **4**. The sequence involves an initial Knoevenagel–cyclocondensation to furnish chalcone intermediates, followed by annulation reactions with hydrazonoyl chlorides, *α*‐bromoketones, and bromomethylcoumarin, as shown in Schemes 1–3. These transformations provided access to structurally diverse triazolo‐ and thiazolopyridopyrimidine derivatives, thereby broadening the potential pharmacological significance of this heterocyclic framework.

The synthetic pathway outlined in Scheme 1 demonstrates the efficiency of a two‐step sequence leading to the target thione derivative **4**. The first step involves a Knoevenagel condensation of 5‐acetyl‐6‐methyl‐4‐phenyl‐3,4‐dihydropyrimidin‐2(1H)‐one (**1**) [58] with benzaldehyde, furnishing the chalcone derivative **2** in good yield. This transformation is facilitated by the activated methylene group of **1**, and the resulting conjugated system **2** serves as an excellent Michael acceptor. The structure of **2** was confirmed by elemental analysis and spectral data (IR, NMR, and MS) (see Experimental section).

Treatment of chalcone **2** with 6‐amino‐2‐thioxo‐1H‐pyrimidin‐4‐one (**3**) yielded the fused heterocyclic compound **4**, identified as 7‐(4‐methyl‐2‐oxo‐6‐phenyl‐1,2,3,4‐tetrahydropyrimidin‐5‐yl)‐5‐phenyl‐2‐thioxo‐2,3‐dihydropyrido[2,3‐d]pyrimidin‐4(1*H*)‐one. The reaction is proposed to occur through nucleophilic attack of the amino group in thiouracil **3** on the activated double bond of chalcone **2**, followed by intramolecular ring closure and subsequent dehydration. This process highlights the value of chalcone–aminothiouracil adducts as key intermediates for assembling condensed pyridopyrimidine frameworks.

The structure of the newly synthesized thione **4** was confirmed by both analytical and spectral data. The absence of the olefinic proton signals from chalcone **2** together with the appearance of characteristic resonances of the thione moiety provided clear evidence of cyclization. IR spectra displayed multiple NH absorptions (3280–3435 cm^−1^), two strong carbonyl bands (1670–1685 cm^−1^), and a C=N stretch at 1605 cm^−1^, consistent with the proposed structure. The ^1^H NMR spectrum showed signals for the methyl group, methine proton, olefinic proton, and three exchangeable NH protons, in addition to aromatic multiplets. Furthermore, the mass spectrum exhibited a molecular ion peak at m/z 441, which agreed with the calculated molecular weight.

The reactivity of thione **4** was further investigated through its interaction with hydrazonoyl chlorides **5a–i** [59, 60, 61] in the presence of triethylamine and dioxane under reflux conditions. The reaction is initiated by nucleophilic substitution at the halogen center, affording the thiohydrazonate intermediates **6**. These intermediates undergo intramolecular cyclization via nucleophilic attack of the adjacent hydrazine nitrogen on the thiocarbonyl carbon, yielding the thiohydrazide intermediates **7**. Subsequent elimination of hydrogen sulfide from **7** furnished the target triazolopyridopyrimidine derivatives **8a–i**. The regiochemical outcome of this transformation is consistent with literature precedents for similar annulation reactions, where triazole ring closure occurs preferentially at the thione site, producing the fully aromatized tricyclic scaffold.

The structures of the newly synthesized compounds **8a–i** were confirmed by a combination of elemental analyses and spectral data. IR spectra demonstrated the disappearance of the thione absorption bands of the precursors and the appearance of new signals corresponding to C=N and triazole ring vibrations, in addition to characteristic NH stretches (∼3330–3445 cm^−1^) and multiple carbonyl absorptions (1660–1730 cm^−1^). The ^1^H NMR spectra revealed the absence of NH signals typical of intermediates **6/7**, along with downfield singlets attributable to triazole protons, as well as resonances for methyl, methine, olefinic, and aromatic protons. Molecular ion peaks observed in the mass spectra matched the calculated molecular weights, with halogenated derivatives exhibiting the expected isotopic patterns. Elemental analyses were consistent with theoretical values, and the compounds were isolated in moderate to good yields (66–79%) (Scheme 2).

The synthetic versatility of thione **4** was further demonstrated by its reaction with *α*‐bromoketones **9a–e** [62] in the presence of anhydrous sodium acetate/dioxane under reflux. The reaction proceeds via initial nucleophilic substitution at the bromine atom, affording the thioether intermediates **10**. Subsequent intramolecular cyclodehydration of **10** furnished the thiazolopyridopyrimidine derivatives **11a–e** (Scheme 3). The regiochemical outcome is consistent with related cyclization reactions, in which the thione group undergoes selective annulation with the bromoacetyl moiety, generating the fused thiazole system.

In a parallel pathway, treatment of compound **4** with 3‐(2‐bromoacetyl)‐2H‐chromen‐2‐one (**12**) under the same conditions yielded the coumarin‐fused thiazolopyridopyrimidine derivative **13** (Scheme 3). This transformation expands the structural diversity of the synthesized heterocycles by incorporating a biologically relevant coumarin nucleus into the pyridopyrimidine framework.

The structures of compounds **11a–e** and **13** were confirmed by elemental analyses and spectral data. In the IR spectra, disappearance of the characteristic thione absorption band, along with the emergence of new bands corresponding to C=N and thiazole functionalities, supported the cyclization process. The ^1^H NMR spectra of the products showed the absence of NH signals characteristic of compound **4**, while new resonances attributable to the thiazole and coumarin moieties were observed. Mass spectral data further corroborated the proposed structures by displaying molecular ion peaks consistent with their molecular formulas.

The spectral and analytical results confirmed the structures of compounds **11a–e**. IR spectra consistently showed NH bands (3370–3445 cm^−1^), two carbonyl absorptions (1660–1680 cm^−1^), and a C=N stretch (∼1605 cm^−1^). In the ^1^H NMR spectra, characteristic signals for the methyl substituents, thiazole H‐5, and the olefinic proton were observed, along with aromatic multiplets reflecting the different substituents. Halogenated derivatives (**11c, 11d**) displayed the expected isotopic patterns in their mass spectra, while elemental analyses agreed with theoretical values. These findings confirm the successful synthesis of the fused heterocyclic systems, obtained in good yields (73–79%).

Overall, these synthetic investigations highlight the efficiency and versatility of the described strategies in accessing a wide range of fused pyridopyrimidine scaffolds. The straightforward Knoevenagel–cyclocondensation pathway furnished novel thione derivatives, which served as key precursors for further annulation. Subsequent transformations with hydrazonoyl chlorides enabled the synthesis of triazolopyridopyrimidines, while reactions with *α*‐bromoketones and bromomethylcoumarin afforded thiazolopyridopyrimidine frameworks with expanded structural diversity. Collectively, these approaches provide convenient and efficient routes to structurally varied fused pyridopyrimidines, thereby broadening their chemical diversity and enhancing their potential biological and pharmacological relevance.

### 2.1 Antitumor Activity

The cytotoxic potential of the synthesized derivatives (**8a–i**, **11a–e**, and **13**) was evaluated against the HEPG2‐1 hepatocellular carcinoma cell line using the MTT assay, with doxorubicin used as the standard reference. Dose–response curves were constructed, and the half‐maximal inhibitory concentrations (IC_50_, µM) were determined, indicating the compound concentration required to suppress 50% of cell proliferation. The reported values represent the mean IC_50_ obtained from three independent experiments (Table 1).

**TABLE 1:** Cytotoxic activity of the tested compounds against HEPG21 cells.

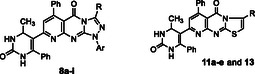			
Compd No.	R	Ar	**IC**_**50,**_µM
**8a**	COCH_3_	Ph	2.73 ± 0.52
**8b**	COCH_3_	4‐CH_3_OC_6_H_4_	1.13 ± 0.31
**8c**	COCH_3_	4‐ClC_6_H_4_	19.27 ± 0.82
**8d**	COOEt	2‐CH_3_C_6_H_4_	0.35 ± 0.31
**8e**	COOEt	3‐CH_3_C_6_H_4_	0.52 ± 0.73
**8f**	COOEt	4‐ClC_6_H_4_	7.14 ± 0.45
**8g**	COOEt	3‐NO_2_C_6_H_4_	9.01 ± 0.47
**8h**	CONHPh	Ph	1.26 ± 0.38
**8i**	CONHPh	2,4‐diClC_6_H_3_	8.35 ± 0.69
**11a**	4‐CH_3_C_6_H_4_	–	3.75 ± 0.54
**11b**	4‐CH_3_OC_6_H_4_	–	1.36 ± 0.42
**11c**	4‐ClC_6_H_4_	–	7.36 ± 0.52
**11d**	4‐BrC_6_H_4_	–	4.14 ± 0.95
**11e**	4‐NO_2_C_6_H_4_	–	9.29 ± 0.50
**13**	3‐Coumarinyl	–	0.46 ± 0.37
**Doxorubicin**	–	–	0.31± 0.48

The cytotoxic evaluation of the synthesized compounds (**8a–i**, **11a–e**, and **13**) against the HEPG21 liver carcinoma cell line revealed a wide activity range, with IC_50_ values spanning from 0.35 to 19.27 µM. Compounds **8d** (0.35 µM), **8e** (0.52 µM), and **13** (0.46 µM) exhibited the most potent activity, nearly comparable to the reference drug doxorubicin (0.31 µM), identifying them as strong lead candidates. Moderate activity was observed for **8b** (1.13 µM), **8hr** (1.26 µM), and **11b** (1.36 µM), while other derivatives, particularly those bearing electron‐withdrawing substituents such as **8c**, **8f**, **8g**, **8i**, **11c**, and **11e**, demonstrated weaker cytotoxic effects with IC_50_ values above 7 µM. Structure–activity relationship analysis suggests that ester functionalities (COOEt) combined with ortho‐ or meta‐substituted aryl groups, as in **8d** and **8e**, enhance potency, while electron‐donating substituents (e.g., OMe in **8b** and **11b**) also improve activity compared to electron‐withdrawing groups (Cl, NO_2_). Notably, the coumarin‐based compound **13** displayed excellent activity, highlighting the beneficial influence of this scaffold. Overall, the results emphasize **8d**, **8e**, and **13** as promising anticancer leads, with activity profiles approaching that of doxorubicin.

### 2.2 In Silico Docking Study

To gain insight into docking scores and molecular interactions, the most potent newly generated ligand molecules and the target enzyme were involved in a molecular docking investigation [63]. Based on the in vitro inhibitory results, compounds **8a, 8b, 8d, 8e, 8hr, 11a–c, 11e,** and **13** are selected as the best and good HepG‐2 inhibitors in this study, as the ligand examples. When introduced into the target enzyme's active site, the most potent synthesized molecules demonstrated related fitness to the reference medication (doxorubicin). Doxorubicin and EGFR are analogous in that they both play important roles in the biology of cancer: EGFR drives tumor development and survival, while doxorubicin disrupts the same pathways. They relate in terms of treatment approaches, particularly in tumors that overexpress EGFR. Doxorubicin was used as an EGFR inhibitor because, according to current research, it may disrupt EGFR/Src signaling [64], which has consequences that expand beyond its traditional DNA‐damaging functions and increase cancer cell death. Fundamental data was exported (Table 2), and docking complex images are presented as well in Figure 2. Docking analyses provided insights into the possible mechanism of action, highlighted by several key findings: (i) binding energy scores (S) were within the range of –8.259 to –9.630 kcal/mol; (ii) the interactions primarily involved the N, S, and C atoms of the methyl group, along with O; (iii) the principal receptor residues engaged in binding were lysine, valine, threonine, asparagine, arginine, leucine, and cysteine; (iv) multiple interaction types were observed, including hydrogen‐bond acceptor, *π*–H, and hydrogen‐bond donor; and (v) collectively, these results suggest that the tested compounds may act as stronger inhibitors of the 3POZ protein than doxorubicin (DOX). The 3D model of the novel ligands **8a** and **8b** gave two H‐bond acceptors between the N of the pyrido[2,3‐*d*]pyrimidin‐4‐one moiety and the residue of Lys745. On the contrary, the docking of **8d** and **11e** gave three H‐bonds: one H‐bond donor between the N of the pyrimidin‐2‐one ring with the essential Asp837 and another two H‐bond acceptors between the N atom of the pyrido[2,3‐*d*]pyrimidin‐4‐one as well as pyrimidin‐2‐one and the residue of Lys745 as well as Arg841, respectively. Additionally, the S atom of **11e** engaged in one hydrogen bond donor with Asp855, while the O atom interacted through hydrogen bonding acceptor with Cys797. The amino group of compounds **8e**, **11a**, **11c,** and **13** formed two hydrogen bonds; one was a donor with Asp837, and another one was an acceptor with Arg841, respectively. In addition, the sulfur atom of **11a**, **11c**, and **13** engaged in one hydrogen bond donor with Asp855. The pyrimidinone fragment was crucial for activity, as it enhanced the potency of **8hr** by forming a hydrogen‐bond donor interaction with Asp800 along with *π*–H contacts. Moreover, the methyl substituent of the pyrimidin‐2‐one ring and the sulfur functional group contributed additional hydrogen‐bond donor interactions with Asp855.

FIGURE 2
3D and 2D ligand interactions for novel synthesized molecules **8a**, **8b**, **8d**, **8e**, **8h**, **11a–c**, **11e**, **13**, and doxorubicin at the 3POZ binding site.

**Figure:**
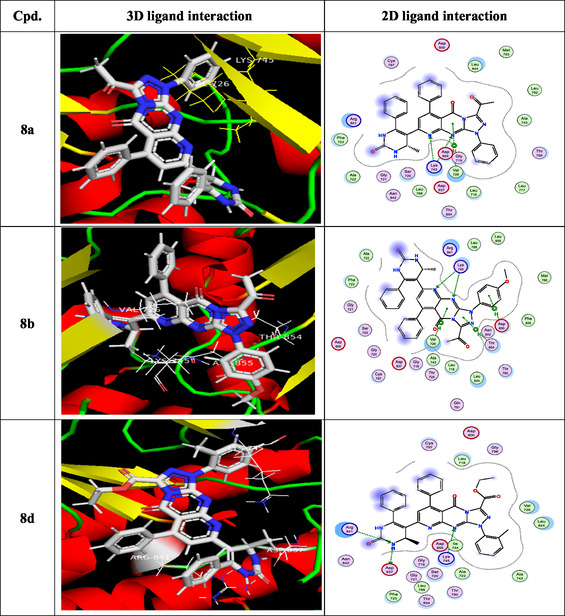

**Figure:**
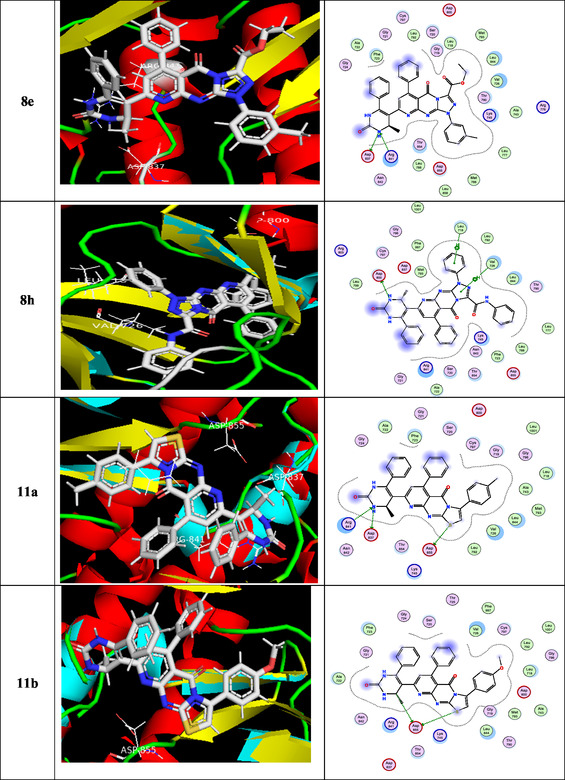

**Figure:**
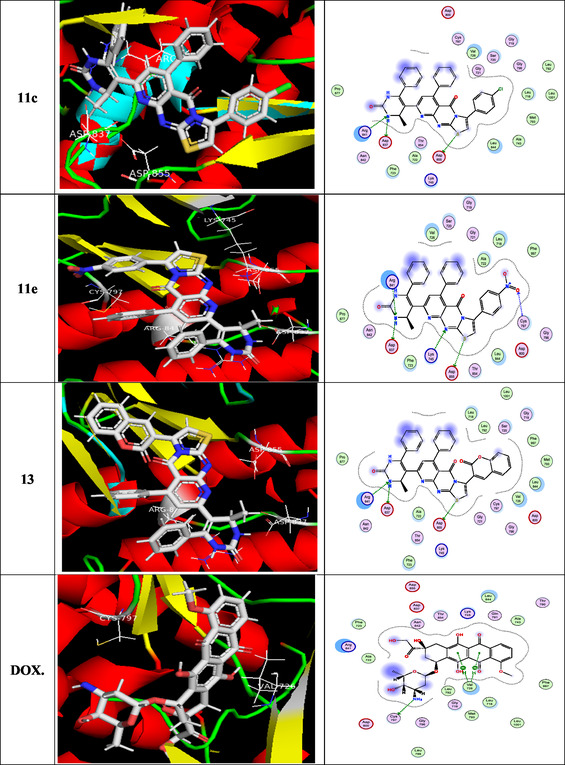

**TABLE 2:** The binding energies (kcal mol^−1^) for docked compounds **8a, 8b, 8d, 8e, 8h, 11a–c, 11e, 13,** and **DOX** at the binding site of the target enzyme.

Compound	Docking score (kcal/mol)	Docked complex (ligand interaction–amino acid)	Donor atom	Acceptor atom
**8a**	−8.765	H—bond: 2 (Lys745) Arene–H: 1 (Val726).	—	N
**8b**	−9.358	H—bond: 2 (Lys745) Arene–H: 1 (Val726) 1 (Thr854) 1 (Asp855).	—	N
**8d**	−9.630	H—bond: 1 (Asp837) 1 (Lys745) 1 (Arg841).	N — —	— N N
**8e**	−9.409	H—bond: 1 (Asp837) 1 (Arg841).	N —	— N
**8h**	−9.422	H—bond: 1 (Asp800) Arene–H: 1 (Leu718) 1 (Val726).	N	—
**11a**	−8.353	H—bond: 1 (Asp855) 1 (Asp837) 1 (Arg841).	S N —	— — N
**11b**	−9.002	H—bond: 2 (Asp855)	S, C	—
**11c**	−8.254	H—bond: 1 (Asp855) 1 (Asp837) 1 (Arg841).	S N —	— — N
**11e**	−8.259	H—bond: 1 (Asp855) 1 (Asp837) 1 (Cys797) 1 (Lys745) 1 (Arg841).	S N — — —	— — O N N
**13**	−9.618	H—bond: 1 (Asp855) 1 (Asp837) 1 (Arg841).	S N —	— — N
**DOX.**	−7.768	H—bond: 1 (Cys797) Arene–H: 2 (Val726).	N	—

Interestingly, all tested compounds achieved more potent docking scores (−8.259 to −9.630 Kcal/mol) than the docking score of crystal reference (−7.768 Kcal/mol).

In summary, the docking analysis confirmed that the investigated compounds achieved strong binding scores and displayed meaningful interaction patterns with the target enzyme, in line with their observed in vitro kinase inhibition. The compounds consistently engaged the receptor through stable binding and comparable interaction modes, with minor differences in orientation. The most favorable docking poses highlighted the formation of stable kinase–ligand complexes, supported by multiple strong and diverse interactions.

### 2.3 Studied Parameters for Drug Likeness and Physicochemical Properties

For drug‐likeness evaluation, several physicochemical parameters are considered. The molecular weight (MW) should typically *lie* between 100 and 600. The number of heteroatoms (nHet), representing noncarbon atoms, is usually optimal in the range of 1–15, while the number of rigid bonds (nRig) is generally between 0 and 30. The largest ring size (MaxRing) is preferred to contain 0–18 atoms, and the total number of rings (nRing) should ideally fall within 0–6. Flexibility is measured by the number of rotatable bonds (nRot), with the acceptable range being 0–11.

Polarity is expressed by the topological polar surface area (TPSA), optimally between 0 and 140. The number of hydrogen bond donors (nHD), calculated from OH and NH groups, is usually 0–7, while the number of hydrogen bond acceptors (nHA), derived from O and N atoms, ranges from 0 to 12. The formal charge (fChar) of drug‐like compounds is typically close to 4. Partitioning properties are described by logP (octanol/water partition coefficient, ideal range 0–3 log mol/L), logD at pH 7.4 (optimal range 1–3 log mol/L), and logS (aqueous solubility, preferred values between –4 and 0.5 log mol/L).

The novel tested compounds exhibit accepted fChar (0), nHet (10–11), and MaxRing (13), which fall within the typical range for drug‐like molecules. Structural rigidity of tested synthesized compounds (7–8 rings, 4–7 rotatable bonds) could influence target binding specificity and metabolic stability. The TPSA varies between 118.6–132.5 Å^2^, with optimal values in tested compounds indicating better potential for passive absorption. The chosen novel compounds exhibited two hydrogen bond donors and nine to eleven hydrogen bond acceptors, as illustrated in Table 3. Also, these compounds contribute to poor aqueous solubility (LogS: −7.67 to −6.83), a potential limitation for oral bioavailability.

**TABLE 3:** Physicochemical properties and drug‐likeness of **8b, 8d, 8e, 13,** and **Dox**.

Cpd.	MW, g/mol	nRig	fChar	nHet	MaxRing	nRing	nRot	TPSA	nHD	nHA	LogD	LogS	Logp
**8b**	597.2	42	0	11	13	7	6	132.5	2	11	4.105	−6.83	5.006
**8d**	611.2	42	0	11	13	7	7	132.5	2	11	4.434	−6.99	5.780
**8e**	611.2	42	0	11	13	7	7	132.5	2	11	4.495	−6.96	5.750
**13**	609.1	47	0	10	13	8	4	118.6	2	9	4.512	−7.67	6.310
**Dox.**	543.1	30	0	12	18	5	5	206.0	7	12	0.738	−3.41	2.012

**FIGURE 3:**
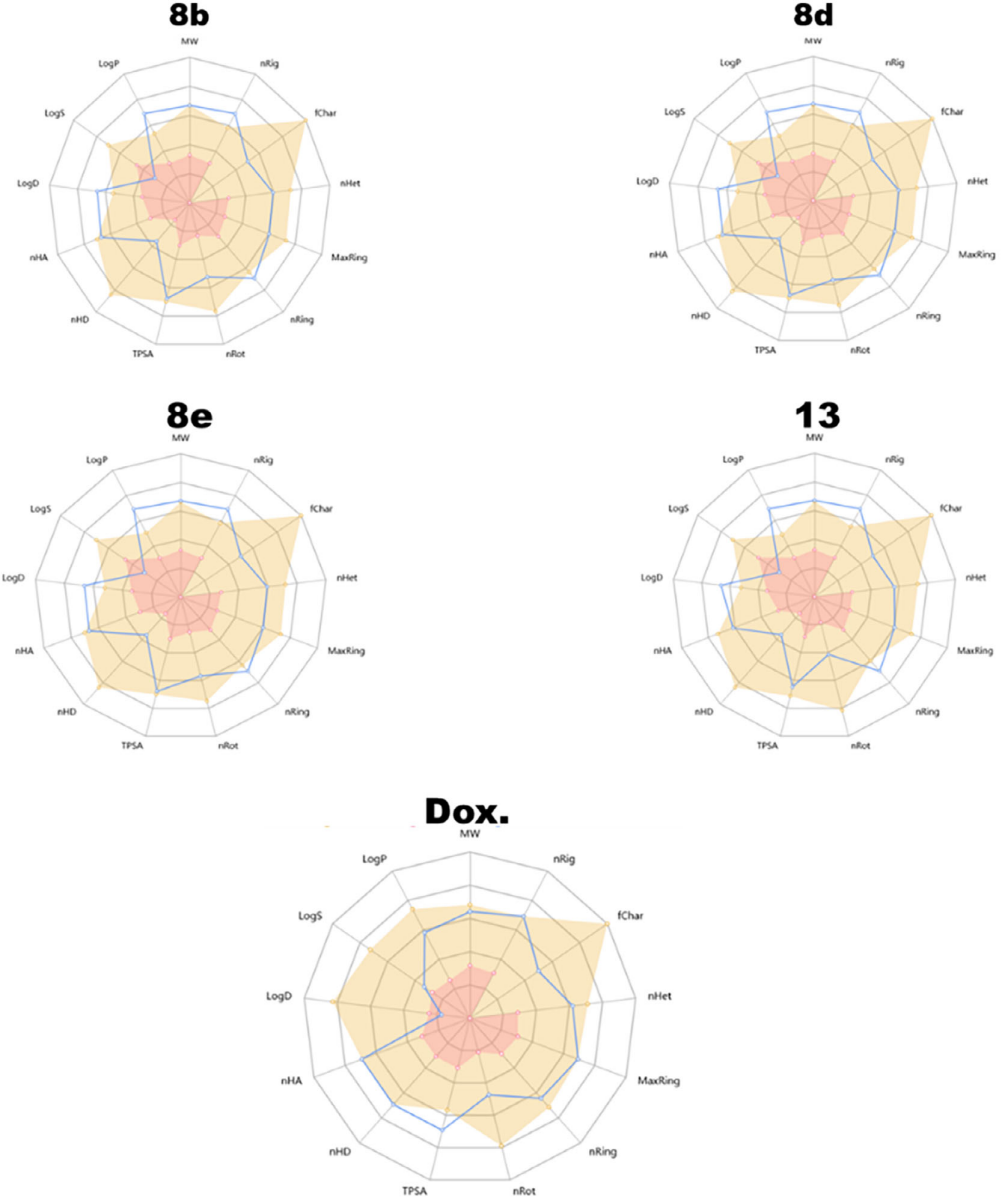
Radar plot of physicochemical and drug‐likeness profiles for **8b, 8d, 8e,** and **13**.

The oral bioavailability graph (Figure 3) likely reflects these challenges, with poor solubility and permeability restricting systemic exposure.

### 2.4 Studied Parameters for ADMET Properties

The evaluation of ADMET profiles for the synthesized compounds included absorption, distribution, metabolism, excretion, and toxicity parameters. **Absorption** was predicted using human intestinal absorption (HIA), where values between 0 and 1 indicate the probability of being HIA positive, with Category 1 assigned to well‐absorbed molecules. **Distribution** was examined through the volume of distribution (VD), classified as high (>15 mL/min/kg), moderate (5–15 mL/min/kg), or low (<5 mL/min/kg). **Metabolic stability** was assessed via cytochrome P450 (CYP2D6), a key phase I enzyme family abundant in the liver and responsible for metabolizing a wide variety of drugs. **Excretion** was evaluated using clearance (CL), where values above 15 mL/min/kg represent high clearance, 5–15 mL/min/kg indicate moderate clearance, and values below 5 mL/min/kg denote low clearance. **Toxicity** was predicted using the Ames mutagenicity assay, with outputs given as negative (non‐mutagenic) or positive (mutagenic). The overall predicted ADMET characteristics of the investigated compounds are summarized in Table 4.

**TABLE 4:** ADMET properties of compounds **8a, 8b, 8d, 8e, 8h, 11a, 11b, 13,** and **Dox**.

Cpd.	Absorption	Distribution	Metabolism	Excretion	Toxicity
	HIA	VD	**Cytochrome P450** **(CYP2D6 inhibitor)**		
				CL	AMES
**8b**	+ve	0.303	‐ve	4.033	‐ve
**8d**	+ve	0.405	‐ve	4.573	‐ve
**8e**	+ve	0.351	‐ve	4.460	‐ve
**13**	+ve	0.421	‐ve	1.598	‐ve
**Dox.**	+ve	1.147	‐ve	17.27	‐ve

Absorption potential of tested compounds appears potential, as evidenced by predicted human intestinal absorption (HIA: 0.007–0.018), compared to the reference drug (0.779). The VD is explicitly reported (0.303–0.421 mL/min/kg); the tested synthesized compounds’ lipophilicity suggests low tissue distribution. Tested compounds strongly inhibit CYP2D6, reducing toxic metabolites. Hepatic clearance rates of tested molecules (1.598–4.573 mL/min/kg). Compounds showed no mutagenicity as predicted by the Ames test used in ADMET Lab 2.0.

## 3 Experimental

### 3.1 Materials and Methods

Melting points were measured on a Gallenkamp IA 9000 digital electrothermal apparatus (Japan) without correction. Infrared spectra were obtained using a Pye‐Unicam SP300 spectrophotometer with samples prepared as KBr pellets. Nuclear magnetic resonance spectra (^1^H at 500 MHz and ^13^C at 125 MHz) were recorded on a JEOL JNM‐ECZR 500 MHz spectrometer. Mass spectra were collected on a Shimadzu GCMS‐Q1000‐EX instrument using electron ionization at 70 eV. Elemental composition (C, H, N, and S) was analyzed with an Elementar Vario LIII CHNS analyzer (Germany) to confirm the purity of the synthesized compounds.

### 3.2 Synthesis of 5‐Cinnamoyl‐4‐Methyl‐6‐Phenyl‐3,4‐Dihydropyrimidin‐2(1H)‐One (2)

A solution of 4‐methyl‐6‐phenyl‐3,4‐dihydropyrimidin‐2(1H)‐one **1** (2.30 g, 100 mmol) in ethanol (30 mL) was treated with benzaldehyde (1.06 g, 100 mmol). To the mixture, aqueous sodium hydroxide (50%, 10 mL) was added dropwise under stirring, and the reaction was allowed to proceed at room temperature for 5–8 h with continuous stirring. The resulting sodium salt of the chalcone was decomposed by addition of ice‐cold 30% HCl. The precipitated chalcone was collected by filtration, washed with water, dried, and recrystallized from ethanol to afford pure chalcone **2**. Yellow solid, mp 198–200°C; yield 63%; IR (KBr): $\bar{V}$ = 3411, 3226 (2NH), 1691, 1665 (2C=O), 1609 (C=N) cm^−1^; ^1^H NMR (DMSO‐*d*_6_): δ = 2.25 (s, 3H, H_3_), 5.44 (s, 1H, CH), 6.90–7.92 (m, 13H, Ar‐H, CH = CH and NH), 7.88, 9.99 (s, D_2_O exchangeable, 1H, NH) ppm; MS (70 eV): *m*/*z* = 318 (M^+^, 73). Anal. Calcd. for C_20_H_18_N_2_O_2_ (318.38): C, 75.45; H, 5.70; N, 8.80%. Found: C, 75.37; H, 5.64; N, 8.71%.

#### 3.2.1 Synthesis of 7‐(4‐Methyl‐2‐Oxo‐6‐Phenyl‐1,2,3,4‐Tetrahydropyrimidin‐5‐yl)‐5‐Phenyl‐2‐Thioxo‐2,3‐Dihydropyrido[2,3‐d]Pyrimidin‐4(1H)‐One (4)

Equimolar amounts of 5‐cinnamoyl‐4‐methyl‐6‐phenyl‐3,4‐dihydropyrimidin‐2(1H)‐one (**2**) (3.18 g, 10 mmol) and 6‐amino‐2‐thiouracil (**3**) (1.43 g, 10 mmol) were heated under reflux in dry DMF (30 mL) for 12 h. After the reaction mixture was cooled to room temperature, the formed precipitate was filtered off, dried, and recrystallized from DMF to afford the pure product. Yellowish‐white solid, mp 313–315°C; yield 75%; IR (KBr): $\bar{V}$ = 3432, 3360, 3311, 3284 (4NH), 1671, 1683 (2C=O), 1605 (C=N) cm^−1^; ^1^H NMR (DMSO‐*d*_6_): δ = 1.87 (s, 3H, CH_3_), 5.39 (s, 1H, CH), 6.75–7.92 (m, 11H, Ar‐H and NH), 8.12 (s, 1H, =CH), 9.93, 11.02, 11.57 (3s, D_2_O exchangeable, 3H, 3NH) ppm; ^13^C NMR (DMSO‐*d*_6_, 125 MHz) δ = 21.3 (CH_3_), 56.1 (CH), 109.5, 117.7, 121.9, 126.7, 126.9, 128.1, 129.0, 132.3, 134.6, 136.4, 144.0, 145.5, 151.3, 155.6 (Ar–C and C=N), 160.1, 168.5, 175.8 (C=O) ppm; MS (70 eV): *m*/*z* = 441 (M^+^, 35). Anal. Calcd. for C_24_H_19_N_5_O_2_S (441.51): C, 65.29; H, 4.34; N, 15.86%. Found: C, 65.08; H, 4.19; N, 15.62%.

### 3.3 General Method for the Synthesis of Pyrido[2,3‐d] [1, 2, 4] Triazolo[4,3‐a]Pyrimidin‐5(1H)‐One Derivatives (8a–i)

A stirred solution of thione 4 (0.441 g, 1 mmol) and the appropriate hydrazonoyl halide 5a–I [59, 60, 61] (1 mmol) in dioxane (10 mL) was treated with triethylamine (0.14 mL, 1 mmol) at room temperature. The mixture was then refluxed for 6–10 h, until TLC monitoring confirmed complete consumption of the starting materials and the release of H_2_S had ceased. Following solvent removal, the residue was triturated with methanol, and the resulting precipitate was collected by filtration. Purification by recrystallization from a suitable solvent furnished the target products 8a–i. The physical properties of the synthesized compounds are provided in the following.

#### 3.3.1 3‐Acetyl‐8‐(4‐Methyl‐2‐Oxo‐6‐Phenyl‐1,2,3,4‐Tetrahydropyrimidin‐5‐Yl)−1,6‐Diphenylpyrido [2,3‐d [1, 2, 4] Triazolo[4,3‐a]Pyrimidin‐5(1H)‐One (8a)

Yellow solid, yield 77%; mp 247–249°C (DMF); IR (KBr): $\bar{V}$ = 3417, 3329 (2NH), 1668, 1679, 1703 (3C=O), 1604 (C=N) cm^−1^; ^1^H NMR (DMSO‐*d*_6_): δ = 2.25 (s, 3H, CH_3_), 2.37 (s, 3H, CH_3_), 5.37 (s, 1H, CH), 6.89–7.73 (m, 15H, Ar‐H), 8.05 (s, 1H, =CH), 9.98, 11.16 (2s, D_2_O exchangeable, 2H, 2NH) ppm; ^13^C NMR (DMSO‐*d*_6_, 125 MHz) δ = 21.0, 24.4 (CH_3_), 56.3 (CH), 112.3, 117.3, 121.1, 123.4, 124.5, 126.4, 126.9, 127.5, 129.1, 129.5, 129.7, 130.6, 134.5, 135.2, 138.4, 140.5, 141.4, 143.5, 149.3, 153.5, 157.6 (Ar–C and C=N), 167.0, 173.7, 193.8 (C=O) ppm; MS (70 eV): *m*/*z* = 567 (M^+^, 19). Anal. Calcd. for C_33_H_25_N_7_O_3_ (567.61): C, 69.83; H, 4.44; N, 17.27. Found: C, 69.76; H, 4.29; N, 17.19%.

#### 3.3.2 3‐Acetyl‐1‐(4‐Methoxyphenyl)‐8‐(4‐Methyl‐2‐Oxo‐6‐Phenyl‐1,2,3,4‐Tetrahydropyrimidin‐5‐yl)‐6‐Phenylpyrido[2,3‐d [1, 2, 4] Triazolo[4,3‐a]Pyrimidin‐5(1H)‐One (8b)

Brown solid, yield 71%; mp 221–223°C (DMF\EtOH); IR (KBr): $\bar{V}$ = 3414, 3362 (2NH), 1667, 1682, 1701 (3C=O), 1611 (C=N) cm^−1^; ^1^H NMR (DMSO‐*d*_6_): δ = 2.26 (s, 3H, CH_3_), 2.37 (s, 3H, CH_3_), 3.77 (s, 3H, OCH_3_), 5.37 (s, 1H, CH), 6.81–7.75 (m, 14H, Ar‐H), 8.12 (s, 1H, =CH), 9.82, 11.02 (2s, D_2_O exchangeable, 2H, 2NH) ppm; MS (70 eV): *m*/*z* = 597 (M^+^, 41). Anal. Calcd. for C_34_H_27_N_7_O_4_ (597.64): C, 68.33; H, 4.55; N, 16.41. Found: C, 68.19; H, 4.48; N, 16.35%.

#### 3.3.3 3‐Acetyl‐1‐(4‐Chlorophenyl)‐8‐(4‐Methyl‐2‐Oxo‐6‐Phenyl‐1,2,3,4‐Tetrahydropyrimidin‐5‐Yl)‐6‐Phenylpyrido[2,3‐d [1, 2, 4] Triazolo[4,3‐a]Pyrimidin‐5(1H)‐One (8c)

Yellow solid, yield 79%; mp 260–262°C (DMF); IR (KBr): $\bar{V}$ = 3419, 3355 (2NH), 1663, 1686, 1707 (3C=O), 1608 (C=N) cm^−1^; ^1^H NMR (DMSO‐*d*_6_): δ = 2.28 (s, 3H, CH_3_), 2.39 (s, 3H, CH_3_), 5.38 (s, 1H, CH), 6.89–7.71 (m, 14H, Ar‐H), 8.09 (s, 1H, =CH), 10.15, 11.06 (2s, D_2_O exchangeable, 2H, 2NH) ppm; MS (70 eV): *m*/*z* = 604 (M^+^ + 2, 11), 602 (M^+^, 37). Anal. Calcd. for C_33_H_24_ClN_7_O_3_ (602.05): C, 65.84; H, 4.02; N, 16.29. Found: C, 65.75; H, 4.14; N, 16.08%.

#### 3.3.4 Ethyl 8‐(4‐Methyl‐2‐Oxo‐6‐Phenyl‐1,2,3,4‐Tetrahydropyrimidin‐5‐Yl)‐5‐Oxo‐6‐Phenyl‐1‐(o‐Tolyl)−1,5‐Dihydropyrido[2,3‐d [1, 2, 4] Triazolo[4,3‐a]Pyrimidine‐3‐Carboxylate (8d)

Yellow solid, yield 70%; mp 203–205°C (EtOH); IR (KBr): $\bar{V}$ = 3429, 3346 (2NH), 1667, 1679, 1728 (3C=O), 1607 (C=N) cm^−1^; ^1^H NMR (DMSO‐*d*_6_): δ = 1.18–1.20 (t, 3H, CH_3_CH_2_), 2.14 (s, 3H, CH_3_), 2.39 (s, 3H, CH_3_), 4.18–4.20 (q, 2H, CH_3_CH_2_), 5.40 (s, 1H, CH), 6.79–7.71 (m, 14H, Ar‐H), 7.98 (s, 1H, =CH), 10.24, 11.02 (2s, D_2_O exchangeable, 2H, 2NH) ppm; MS (70 eV): *m*/*z* = 611 (M^+^, 72). Anal. Calcd. for C_35_H_29_N_7_O_4_ (611.66): C, 68.73; H, 4.78; N, 16.03. Found: C, 68.61; H, 4.65; N, 16.01%.

#### 3.3.5 Ethyl 8‐(4‐Methyl‐2‐Oxo‐6‐Phenyl‐1,2,3,4‐Tetrahydropyrimidin‐5‐Yl)‐5‐Oxo‐6‐Phenyl‐1‐(m‐Tolyl)−1,5‐Dihydropyrido[2,3‐d [1, 2, 4] Triazolo[4,3‐a]Pyrimidine‐3‐Carboxylate (8e)

Yellow solid, yield 69%; mp 185–187°C (EtOH); IR (KBr): $\bar{V}$ = 3408, 3330 (2NH), 1667, 1681, 1727 (3C=O), 1607 (C=N) cm^−1^; ^1^H NMR (DMSO‐*d*_6_): δ = 1.18–1.21 (t, 3H, CH_3_CH_2_), 2.29 (s, 3H, CH_3_), 2.39 (s, 3H, CH_3_), 4.21–4.23 (q, 2H, CH_3_CH_2_), 5.34 (s, 1H, CH), 6.91–7.75 (m, 14H, Ar‐H), 7.92 (s, 1H, =CH), 10.17, 11.65 (2s, D_2_O exchangeable, 2H, 2NH) ppm; MS (70 eV): *m*/*z* = 611 (M^+^, 35). Anal. Calcd. for C_35_H_29_N_7_O_4_ (611.66): C, 68.73; H, 4.78; N, 16.03. Found: C, 68.63; H, 4.69; N, 15.97%.

#### 3.3.6 Ethyl 1‐(4‐Chlorophenyl)‐8‐(4‐Methyl‐2‐Oxo‐6‐Phenyl‐1,2,3,4‐Tetrahydropyrimidin‐5‐Yl)‐5‐Oxo‐6‐Phenyl‐1,5‐Dihydropyrido[2,3‐d [1, 2, 4] Triazolo[4,3‐a]Pyrimidine‐3‐Carboxylate (8f)

Yellow solid, yield 73%; mp 217–219°C (EtOH); IR (KBr): $\bar{V}$ = 3423, 3351 (2NH), 1670, 1687, 1727 (3C=O), 1607 (C=N) cm^−1^; ^1^H NMR (DMSO‐*d*_6_): δ = 1.29–1.32 (t, 3H, CH_3_CH_2_), 2.29 (s, 3H, CH_3_), 4.23–4.25 (q, 2H, CH_3_CH_2_), 5.39 (s, 1H, CH), 6.68–7.72 (m, 14H, Ar‐H), 8.12 (s, 1H, =CH), 9.02, 10.75 (2s, D_2_O exchangeable, 2H, 2NH) ppm; ^13^C NMR (DMSO‐*d*_6_, 125 MHz) δ = 13.5, 21.7 (CH_3_), 56.7(CH), 61.9 (CH_2_), 112.2, 116.1, 118.6, 119.2, 120.2, 121.6, 121.9, 124.0, 124.7, 128.7, 129.1, 129.6, 134.1, 136.8, 138.5, 138.9, 139.3, 139.5, 142.6, 146.3, 153.9 (Ar–C and C=N), 160.0, 163.4, 174.0 (C=O) ppm; MS (70 eV): *m*/*z* = 634 (M^+^ + 2, 19), 632 (M^+^, 62). Anal. Calcd. for C_34_H_26_ClN_7_O_4_ (632.08): C, 64.61; H, 4.15; N, 15.51. Found: C, 64.53; H, 4.12; N, 15.38%.

#### 3.3.7 Ethyl 8‐(4‐Methyl‐2‐Oxo‐6‐Phenyl‐1,2,3,4‐Tetrahydropyrimidin‐5‐Yl)‐1‐(3‐Nitrophenyl)‐5‐Oxo‐6‐Phenyl‐1,5‐Dihydropyrido[2,3‐d [1, 2, 4] Triazolo[4,3‐a]Pyrimidine‐3‐Carboxylate (8g)

Brown solid, yield 66%; mp 238–240°C (DMF); IR (KBr): $\bar{V}$ = 3447, 3359 (2NH), 1671, 1683, 1729 (3C=O), 1609 (C=N) cm^−1^; ^1^H NMR (DMSO‐*d*_6_): δ = 1.24–1.27 (t, 3H, CH_3_CH_2_), 2.34 (s, 3H, CH_3_), 4.21–4.23 (q, 2H, CH_3_CH_2_), 5.39 (s, 1H, CH), 6.91–7.79 (m, 14H, Ar‐H), 8.13 (s, 1H, =CH), 9.32, 10.63 (2s, D_2_O exchangeable, 2H, 2NH) ppm; MS (70 eV): *m*/*z* = 642 (M^+^, 39). Anal. Calcd. for C_34_H_26_N_8_O_6_ (642.63): C, 63.55; H, 4.08; N, 17.44. Found: C, 63.37; H, 4.00; N, 17.31%.

#### 3.3.8 8‐(4‐Methyl‐2‐Oxo‐6‐Phenyl‐1,2,3,4‐Tetrahydropyrimidin‐5‐Yl)‐5‐Oxo‐N,1,6‐Triphenyl‐1,5‐Dihydropyrido[2,3‐d [1, 2, 4] Triazolo[4,3‐a]Pyrimidine‐3‐Carboxamide (8h)

Yellow solid, yield 76%; mp 256–258°C (DMF); IR (KBr): $\bar{V}$ = 3436, 3348, 3297 (3NH), 1661, 1674, 1681 (3C=O), 1604 (C=N) cm^−1^; ^1^H NMR (DMSO‐*d*_6_): δ = 2.21 (s, 3H, CH_3_), 5.32 (s, 1H, CH), 6.91–7.75 (m, 20H, Ar‐H), 8.10 (s, 1H, =CH), 9.13, 10.17, 11.69 (3s, D_2_O exchangeable, 3H, 3NH) ppm; ^13^C NMR (DMSO‐*d*_6_, 125 MHz) δ = 21.1 (CH_3_), 56.5 (CH), 112.1, 115.2, 119.1, 120.0, 121.5, 123.8, 124.1, 127.7, 128.1, 129.2, 129.4, 129.6, 129.9, 136.3, 138.3, 138.4, 138.8, 139.2, 140.0, 141.9, 142.7, 145.2, 146.8, 153.8, 157.3 (Ar–C and C=N), 163.9, 166.9, 173.1 (C=O) ppm; MS (70 eV): *m*/*z* = 644 (M^+^, 100). Anal. Calcd. for C_38_H_28_N_8_O_3_ (644.70): C, 70.80; H, 4.38; N, 17.38. Found: C, 70.73; H, 4.31; N, 17.29%.

#### 3.3.9 1‐(2,4‐Dichlorophenyl)‐8‐(4‐Methyl‐2‐Oxo‐6‐Phenyl‐1,2,3,4‐Tetrahydropyrimidin‐5‐Yl)‐5‐Oxo‐N, 6‐Diphenyl‐1,5‐Dihydropyrido[2,3‐d [1, 2, 4] Triazolo[4,3‐a]Pyrimidine‐3‐Carboxamide (8i)

Yellow solid, yield 77%; mp 271–273°C (DMF); IR (KBr): $\bar{V}$ = 3440, 3371, 3319 (3NH), 1662, 1670, 1683 (3C=O), 1607 (C=N) cm^−1^; ^1^H NMR (DMSO‐*d*_6_): δ = 2.24 (s, 3H, CH_3_), 5.13 (s, 1H, CH), 6.92–7.83 (m, 18H, Ar‐H), 8.14 (s, 1H, =CH), 9.17, 10.21, 11.68 (3s, D_2_O exchangeable, 3H, 3NH) ppm; MS (70 eV): *m*/*z* = 713 (M^+^, 100). Anal. Calcd. for C_38_H_26_Cl_2_N_8_O_3_ (713.58): C, 63.96; H, 3.67; N, 15.70. Found: C, 63.82; H, 3.55; N, 15.59%.

### 3.4 Reactions of Thione 4 with α‐Bromoketones (9a–f, 12)

A mixture of thione **4** (0.441 g, 1 mmol) and the appropriate *α*‐bromoketone **9a–e** [62] (1 mmol) in dioxane (30 mL) containing anhydrous sodium acetate (0.1 g, 1 mmol) was refluxed for 6–10 h, with progress monitored by TLC. The product gradually precipitated during the reaction. The solid was collected by filtration, washed with water, dried, and recrystallized from ethanol to afford the corresponding derivatives **11a–e** and **13**,

#### 3.4.1 8‐(4‐Methyl‐2‐Oxo‐6‐Phenyl‐1,2,3,4‐Tetrahydropyrimidin‐5‐Yl)‐6‐Phenyl‐3‐(p‐Tolyl)‐5H‐Pyrido[2,3‐d]Thiazolo[3,2‐a]Pyrimidin‐5‐One (11a)

Yellow solid, yield 78%; mp 237–239°C (DMF\EtOH); IR (KBr): $\bar{V}$ = 3443, 3352 (2NH), 1669, 1678 (2C=O), 1607 (C=N) cm^−1^; ^1^H NMR (DMSO‐*d*_6_): δ = 2.11 (s, 3H, CH_3_), 2.37 (s, 3H, CH_3_), 5.39 (s, 1H, CH), 6.82 (s, 1H, thiazole‐H5), 7.13–7.83 (m, 14H, Ar‐H), 8.06 (s, 1H, =CH), 9.18, 10.47 (2s, D_2_O exchangeable, 2H, 2NH) ppm; ^13^C NMR (DMSO‐*d*_6_, 125 MHz) δ = 21.0, 22.7 (CH_3_), 55.9 (CH), 109.4, 112.5, 115.0, 120.2, 123.0, 123.8, 125.3, 126.2, 126.8, 128.3, 128.9, 129.2, 129.8, 131.6, 133.6, 135.0, 137.8, 139.0, 141.4, 143.0, 147.8, 153.1(Ar–C and C=N), 167.3, 173.7 (C=O) ppm; MS (70 eV): *m*/*z* = 555 (M^+^, 47). Anal. Calcd. for C_33_H_25_N_5_O_2_S (555.66): C, 71.33; H, 4.54; N, 12.60. Found: C, 71.21; H, 4.48; N, 12.53%.

#### 3.4.2 3‐(4‐Methoxyphenyl)‐8‐(4‐Methyl‐2‐Oxo‐6‐Phenyl‐1,2,3,4‐Tetrahydropyrimidin‐5‐Yl)‐6‐Phenyl‐5H‐Pyrido[2,3‐d]Thiazolo[3,2‐a]Pyrimidin‐5‐One (11b)

Yellow solid, yield 73%; mp 212–214°C (EtOH); IR (KBr): $\bar{V}$ = 3436, 3371 (2NH), 1662, 1675 (2C=O), 1606 (C=N) cm^−1^; ^1^H NMR (DMSO‐*d*_6_): δ = 2.10 (s, 3H, CH_3_), 3.81 (s, 3H, OCH_3_), 5.37 (s, 1H, CH), 6.73 (s, 1H, thiazole‐H5), 6.93–7.87 (m, 14H, Ar‐H), 7.99 (s, 1H, =CH), 9.11, 10.39 (2s, D_2_O exchangeable, 2H, 2NH) ppm; MS (70 eV): *m*/*z* = 571 (M^+^, 60). Anal. Calcd. for C_33_H_25_N_5_O_3_S (571.66): C, 69.34; H, 4.41; N, 12.25. Found: C, 69.25; H, 4.27; N, 12.18%.

#### 3.4.3 3‐(4‐Chlorophenyl)‐8‐(4‐Methyl‐2‐Oxo‐6‐Phenyl‐1,2,3,4‐Tetrahydropyrimidin‐5‐Yl)‐6‐Phenyl‐5H‐Pyrido[2,3‐d]Thiazolo[3,2‐a]Pyrimidin‐5‐One (11c)

Yellow solid, yield 77%; mp 259–261°C (DMF); IR (KBr): $\bar{V}$ = 3442, 3382 (2NH), 1666, 1679 (2C=O), 1604 (C=N) cm^−1^; ^1^H NMR (DMSO‐*d*_6_): δ = 2.11 (s, 3H, CH_3_), 5.42 (s, 1H, CH), 6.72 (s, 1H, thiazole‐H5), 6.91–7.61 (m, 14H, Ar‐H), 8.13 (s, 1H, =CH), 9.12, 10.39 (2s, D_2_O exchangeable, 2H, 2NH) ppm; MS (70 eV): m/*z* = 578 (M^+^ + 2, 7), 576 (M^+^, 23). Anal. Calcd. for C_32_H_22_ClN_5_O_2_S (576.07): C, 66.72; H, 3.85; N, 12.16. Found: C, 66.60; H, 3.78; N, 12.07%.

#### 3.4.4 3‐(4‐Bromophenyl)‐8‐(4‐Methyl‐2‐Oxo‐6‐Phenyl‐1,2,3,4‐Tetrahydropyrimidin‐5‐Yl)‐6‐Phenyl‐5H‐Pyrido[2,3‐d]Thiazolo[3,2‐a]Pyrimidin‐5‐One (11d)

Yellow solid, yield 77%; mp 259–261°C (DMF); IR (KBr): $\bar{V}$ = 3433, 3373 (2NH), 1663, 1675 (2C=O), 1608 (C=N) cm^−1^; ^1^H NMR (DMSO‐*d*_6_): δ = 2.11 (s, 3H, CH_3_), 5.41 (s, 1H, CH), 6.81 (s, 1H, thiazole‐H5), 7.06–7.92 (m, 14H, Ar‐H), 8.01 (s, 1H, =CH), 9.11, 10.39 (2s, D_2_O exchangeable, 2H, 2NH) ppm; MS (70 eV): m/*z* = 622 (M^+^ + 2, 30), 620 (M^+^, 33). Anal. Calcd. for C_32_H_22_BrN_5_O_2_S (620.53): C, 61.94; H, 3.57; N, 11.29. Found: C, 61.83; H, 3.46; N, 11.17%.

#### 3.4.5 8‐(4‐Methyl‐2‐Oxo‐6‐Phenyl‐1,2,3,4‐Tetrahydropyrimidin‐5‐Yl)‐3‐(4‐Nitrophenyl)‐6‐Phenyl‐5H‐Pyrido[2,3‐d]Thiazolo[3,2‐a]Pyrimidin‐5‐One (11e)

Brown solid, yield 79%; mp 202–204°C (DMF); IR (KBr): $\bar{V}$ = 3442, 3382 (2NH), 1666, 1679 (2C=O), 1609 (C=N) cm^−1^; ^1^H NMR (DMSO‐*d*_6_): δ = 1.73 (s, 3H, CH_3_), 5.46 (s, 1H, CH), 6.98 (s, 1H, thiazole‐H5), 7.12–8.22 (m, 14H, Ar‐H), 8.01 (s, 1H, =CH), 9.31, 10.44 (2s, D_2_O exchangeable, 2H, 2NH) ppm; MS (70 eV): m/*z* = 583 (M^+^, 73). Anal. Calcd. for C_32_H_22_N_6_O_4_S (586.63): C, 65.52; H, 3.78; N, 14.33. Found: C, 65.47; H, 3.69; N, 14.27%.

#### 3.4.6 8‐(4‐Methyl‐2‐Oxo‐6‐Phenyl‐1,2,3,4‐Tetrahydropyrimidin‐5‐Yl)‐3‐(2‐Oxo‐2H‐Chromen‐3‐Yl)‐6‐Phenyl‐5H‐Pyrido[2,3‐d]Thiazolo[3,2‐a]Pyrimidin‐5‐One (13)

Yellow solid, yield 74%; mp 249–251°C (DMF); IR (KBr): $\bar{V}$ = 3448, 3357 (2NH), 1665, 1675, 1692 (3C=O), 1609 (C=N) cm^−1^; ^1^H NMR (DMSO‐*d*_6_): δ = 2.13 (s, 3H, CH_3_), 5.41 (s, 1H, CH), 6.79 (s, 1H, thiazole‐H5), 7.05–7.97 (m, 15H, Ar‐H), 8.06 (s, 1H, =CH), 9.27, 10.65 (2s, D_2_O exchangeable, 2H, 2NH) ppm; MS (70 eV): m/*z* = 609 (M^+^, 83). Anal. Calcd. for C_35_H_23_N_5_O_4_S (609.66): C, 68.95; H, 3.80; N, 11.49. Found: C, 68.80; H, 3.69; N, 11.38%.

### 3.5 Anticancer Activity

The cytotoxic activity of the synthesized compounds was evaluated according to the procedure described in reference [64].

#### 3.5.1 Docking Study

##### 3.5.1.1 Ligand preparation

Compounds showing the most potent and moderate IC_50_ values in the MTT assay were chosen for docking experiments. Their chemical structures were generated with ChemDraw Professional 16.0 and optimized using the MMFF94× force field until convergence at an RMSD gradient of 0.1 kcal·mol^−1^·Å^−1^ [65].

##### 3.5.1.2 Protein preparation

The crystal structure of the target enzyme (PDB ID: 3POZ, resolution 1.50 Å) was retrieved from the Protein Data Bank [66]. Before docking, the structure was refined by removing water molecules, adding hydrogen atoms with appropriate geometry, correcting bonds, and minimizing potential energy. The binding site was identified with Alpha Site Finder, and the defined pocket was stored in MOE format for subsequent simulations [67]. Docking was carried out using the triangle matcher algorithm with the London dG scoring function [68]. The ligand–protein complexes were then examined for interactions with key amino acid residues in both 2D and 3D views. All computational procedures were conducted in accordance with standard docking methodologies. [67, 69, 70, 71]

### 3.6 Drug Likeness and ADMET Properties

Evaluating both physicochemical and ADMET properties is essential for assessing potential drug candidates [72]. The online platform Admetlab 2.0 provides a systematic approach for predicting ADMET parameters along with key physicochemical descriptors [73, 74]. Using this tool, the physicochemical profiles, drug‐likeness, and ADMET properties of selected compounds (**8b**, **8d**, **8e**, and **13**) were investigated.

## 4 Conclusion

In this work, a novel series of fused pyridopyrimidine scaffolds was efficiently synthesized through versatile annulation strategies. The synthetic routes provided straightforward access to structurally diverse thiazolo‐ and triazolopyridopyrimidine derivatives, including coumarin‐fused analogs, thereby broadening the chemical diversity of this heterocyclic framework. The biological evaluation of the synthesized compounds against HepG2 cells revealed promising cytotoxic activity, with compounds **8d**, **8e**, and **13** displaying IC_50_ values in the submicromolar range, comparable to doxorubicin. Structure–activity relationship analysis indicated that ester functionalities and coumarin incorporation enhanced potency, while electron‐donating aryl substituents also improved activity. Molecular docking studies supported these findings, showing strong binding affinities of the active compounds toward the EGFR kinase domain with docking scores superior to doxorubicin. Key hydrogen‐bonding and hydrophobic interactions with residues Lys745, Asp837, Arg841, and Asp855 were identified as critical for binding. Furthermore, in silico ADMET predictions suggested favorable drug‐likeness profiles, with acceptable absorption, metabolic stability, and nonmutagenic character, despite some limitations in solubility.

Collectively, the combined synthetic, biological, and computational studies highlight compounds **8d**, **8e**, and **13** as promising lead candidates for further optimization as EGFR‐targeted anticancer agents. These findings reinforce the potential of pyridopyrimidine‐based scaffolds as versatile platforms for anticancer drug discovery.

## Supporting Information

Additional supporting information can be found online in the Supporting Information section.

## Author Contributions

**Sobhi M. Gomha**: conceptualization (lead), data curation (lead), investigation (equal), methodology (lead), writing – original draft (lead), writing – review and editing (equal). **Sami A. Al‐Hussain:** methodology (supporting), investigation – biological studies (lead), formal analysis (equal), writing – review and editing (supporting), funding acquisition (supporting), project administration (supporting). **Basant Farag:** methodology (supporting), investigation (equal), spectroscopic characterization (equal), biological testing (equal), writing – original draft (supporting). **AbdElAziz A. Nayl:** synthetic investigation (supporting), molecular docking and computational analysis (lead), writing – original draft (supporting). **Wesam Hussein:** synthetic investigation (supporting), molecular docking (supporting), data curation (supporting). **Abdelwahed R. Sayed:** spectroscopic characterization (equal), datacuration (supporting). **Magdi E. A. Zaki:** conceptualization (equal), formal analysis (equal), writing – review and editing (lead), supervision (equal), project administration (lead), funding acquisition (lead).

## Funding

This work was supported by the Imam Mohammad Ibn Saud Islamic University (Grant: IMSIU‐DDRSP2501).

## Conflicts of Interest

The authors declare no conflicts of interest.

## Supporting information

Supplementary Material

## Data Availability

The data that support the findings of this study are available from the corresponding author upon reasonable request.
