# Supplementary material for: Synthesis of Pyrido[2,3‐d]Azolopyrimidinones: Design and Epidermal Growth Factor Receptor‐Targeted Molecular Docking Toward Novel Anticancer Leads
Source: ChemistryOpen. 2025 Nov 23;15(4):e202500555. doi: 10.1002/open.202500555 (PMC13051936; doi:10.1002/open.202500555)
Supplement: Supplementary file 1 — Supplementary Material [file OPEN-15-e202500555-s001.pdf]

Supplementary material file

Synthesis of Pyrido[2,3-d]azolopyrimidinones: Design and Epidermal Growth Factor Receptor-Targeted Molecular Docking toward Novel Anticancer Leads.

Sobhi M. Gomha[1]*, Sami A. Al-Hussain[2], Basant Farag[3], AbdElAziz A. Nayl[4], Wesam Hussein[1], Abdelwahed R. Sayed[5], Magdi E. A. Zaki[2]*

[1] Department of Chemistry, Faculty of Science, Islamic University of Madinah, Madinah, 42351, Saudi Arabia, smgomha@iu.edu.sa (SG), 400750@iu.edu.sa (WH),
[2] Department of Chemistry, Faculty of Science, Imam Mohammad Ibn Saud Islamic University (IMSIU), Riyadh 11623, Saudi Arabia; sahussain@imamu.edu.sa (SA), mezaki@imamu.edu.sa (MZ)
[3] Department of Chemistry, Faculty of Science, Zagazig University, Zagazig 44519, Egypt. basantfarag@zu.edu.eg
[4] Department of Chemistry, College of Science, Jouf University, Sakaka 72341, Al Jouf, Saudi Arabia; aanayel@ju.edu.sa
[5]Department of Chemistry, Faculty of Science, University of Beni Suef, Egypt; arashad_2003@yahoo.com

* **Corresponding authors:** mezaki@imamu.edu.sa (MZ), smgomha@iu.edu.sa (SG)

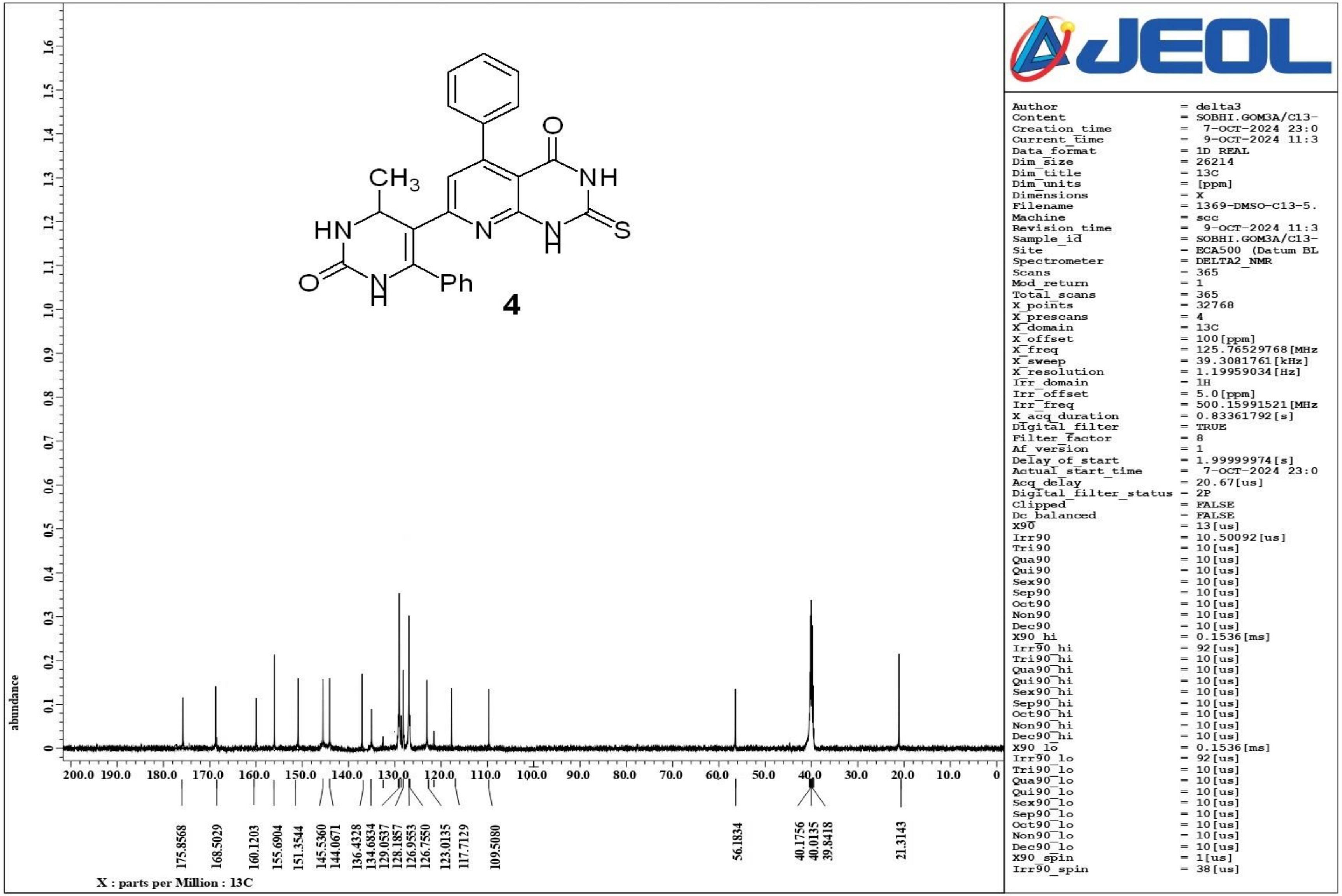

^{13}C-NMR spectra of compound **4**

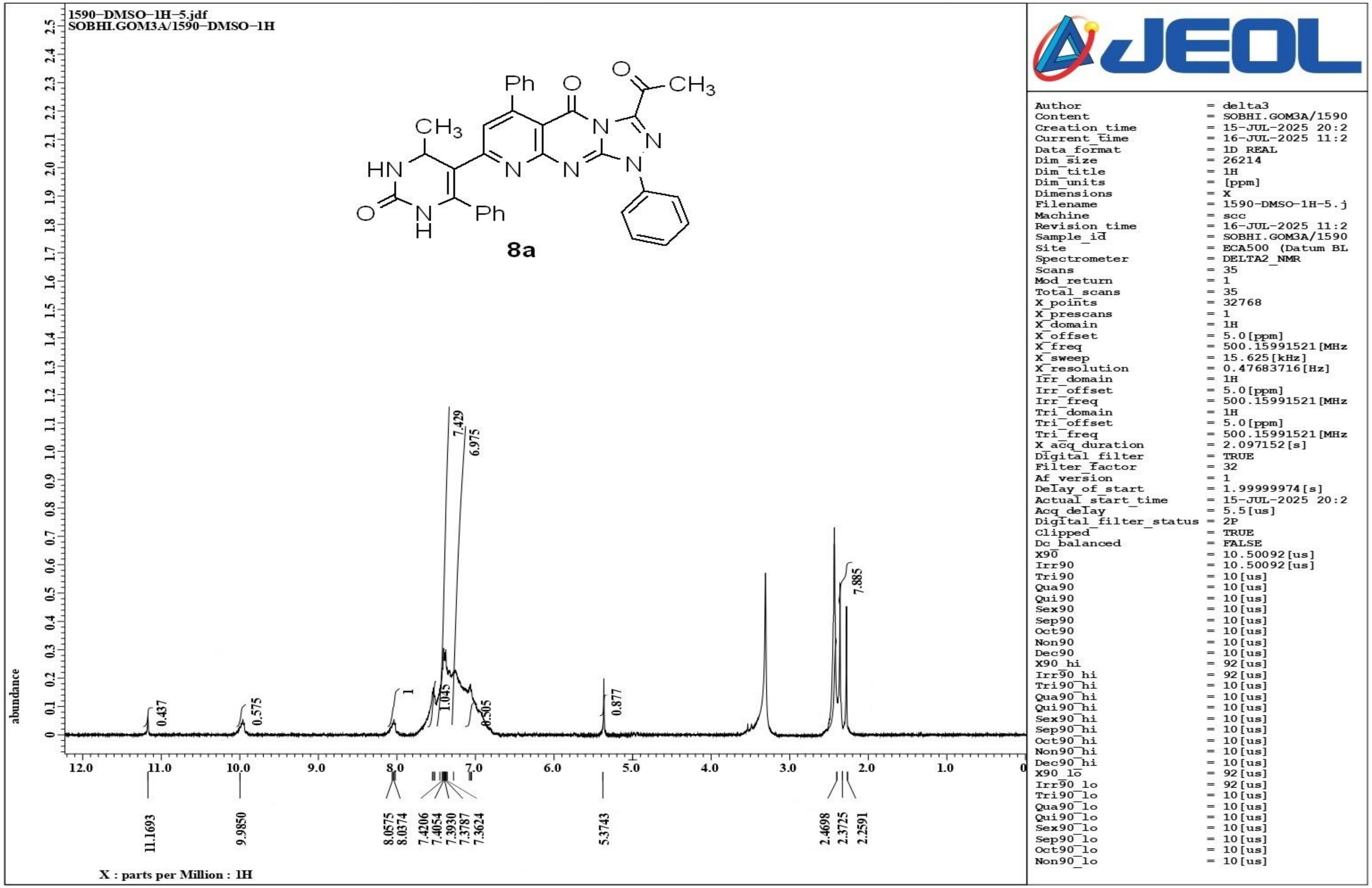

^{1}H-NMR spectra of compound **8a**

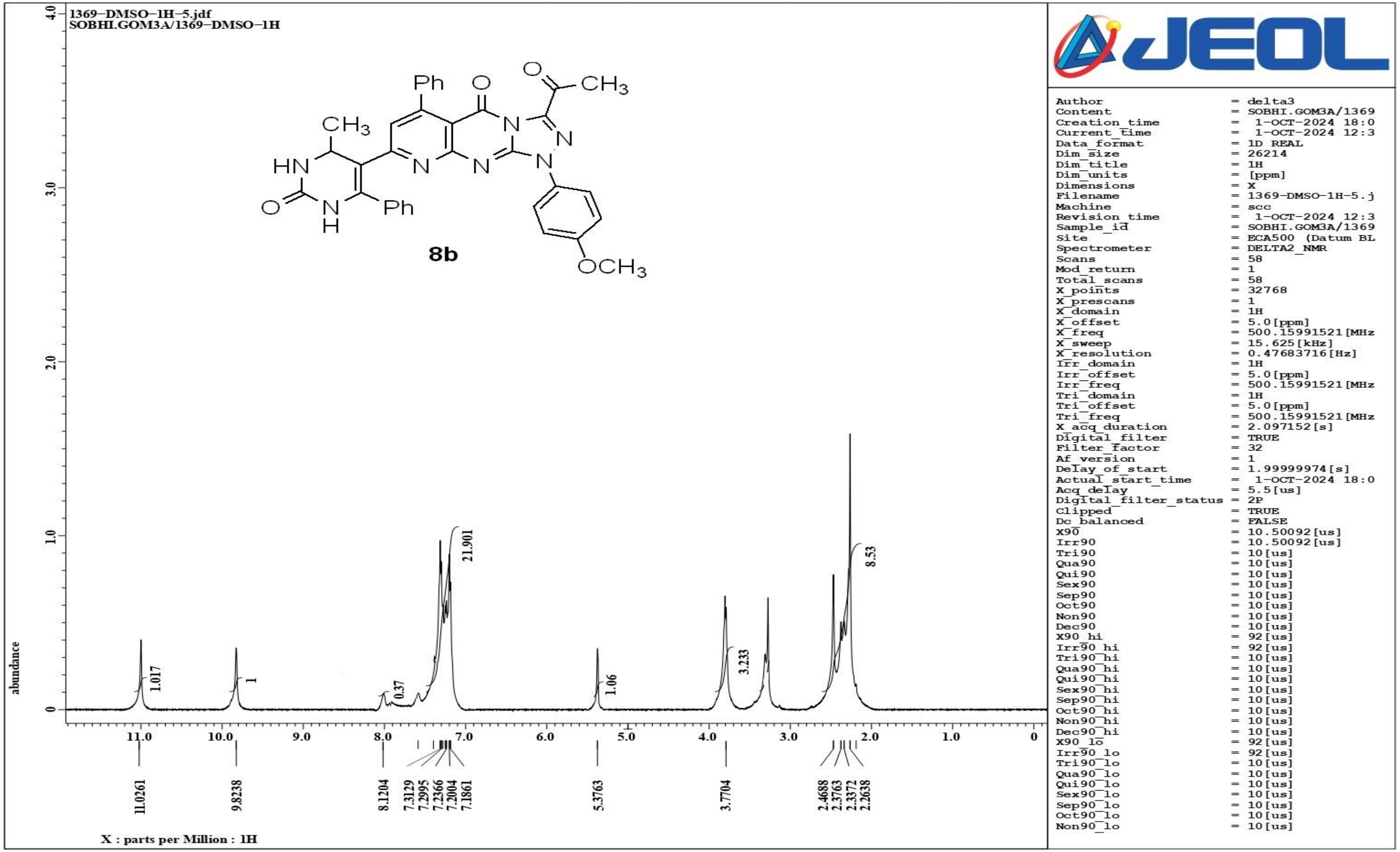

^{1}H-NMR spectra of compound **8b**

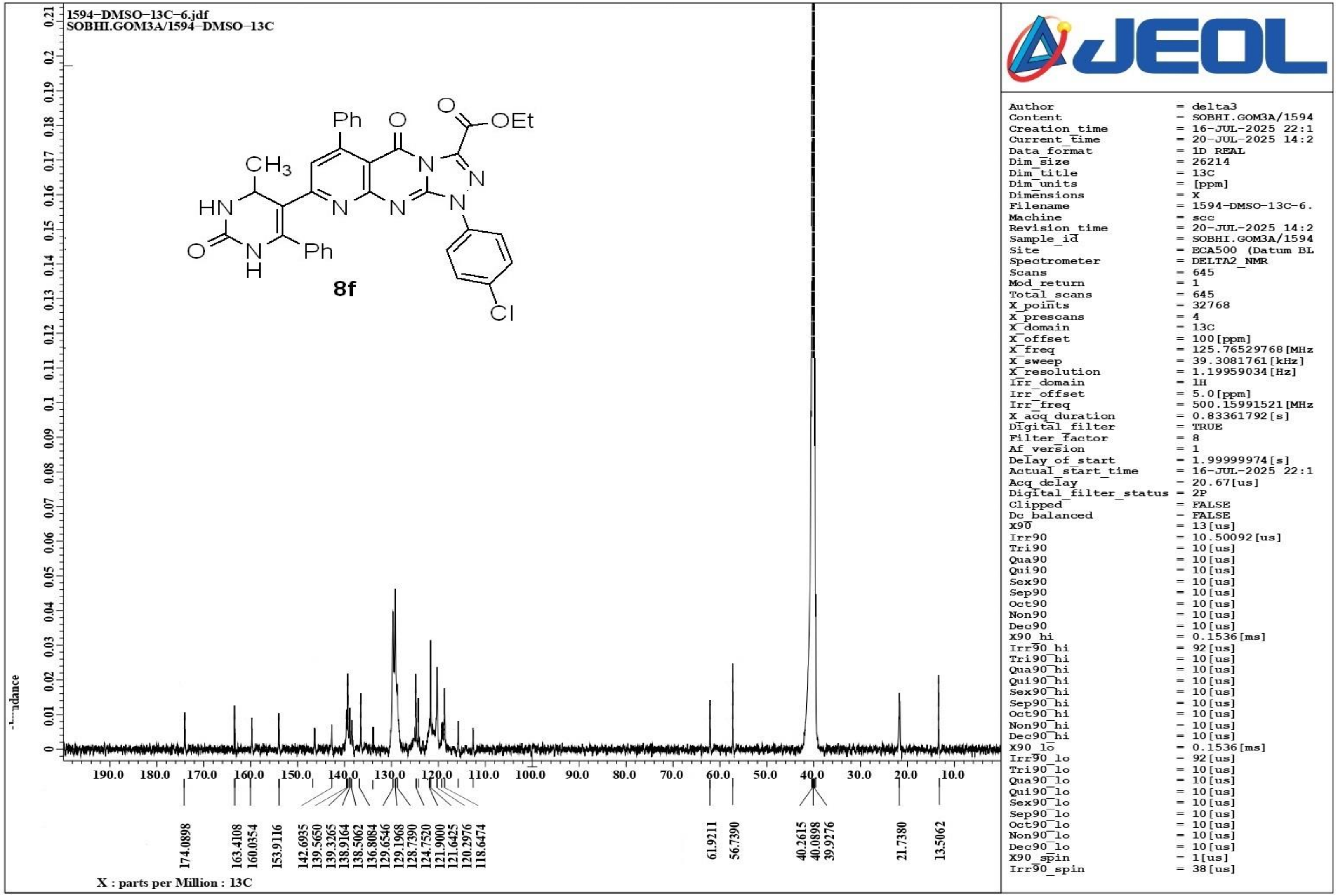

^{13}C-NMR spectra of compound **8f**

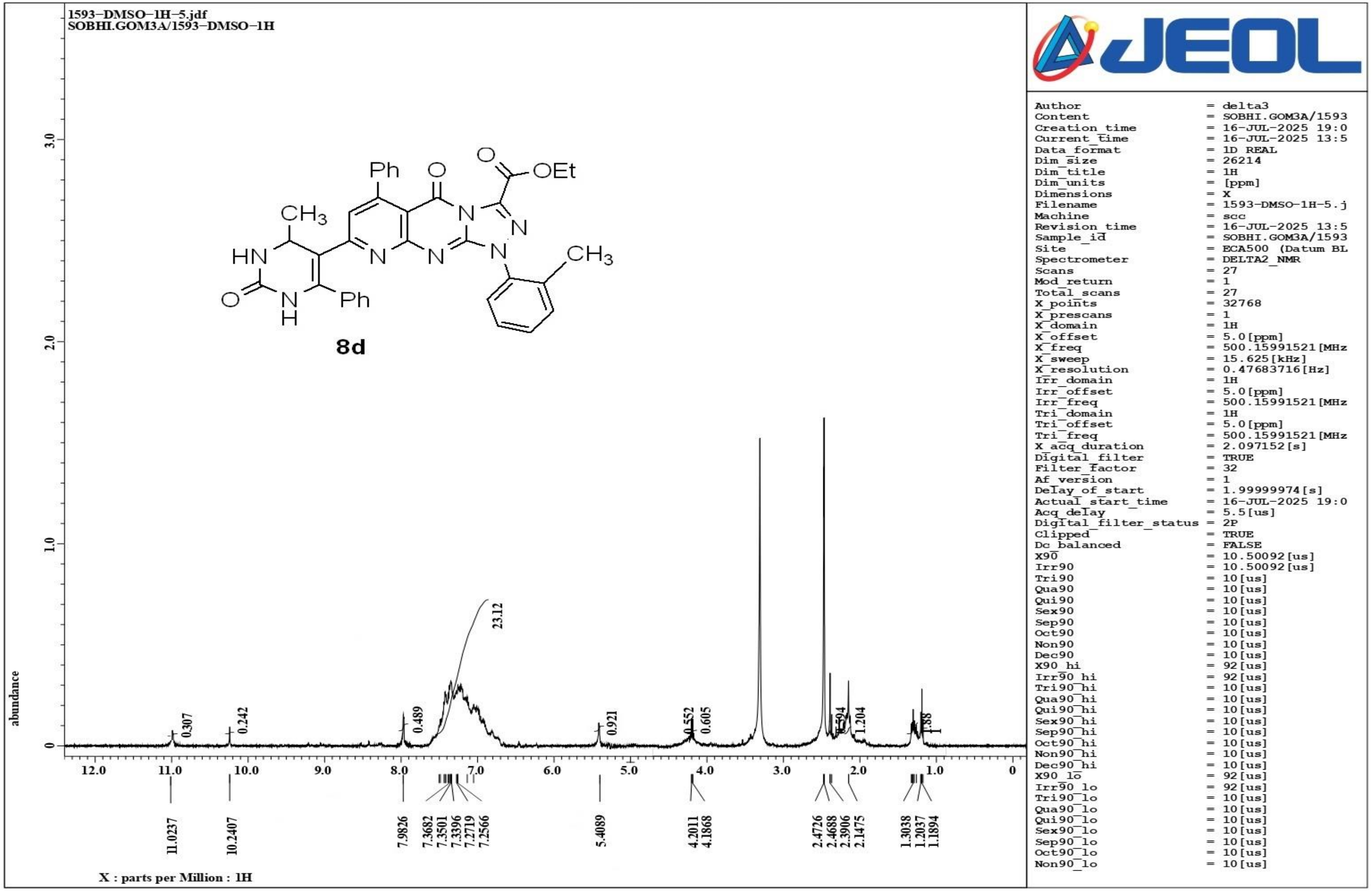

^{1}H-NMR spectra of compound **8d**

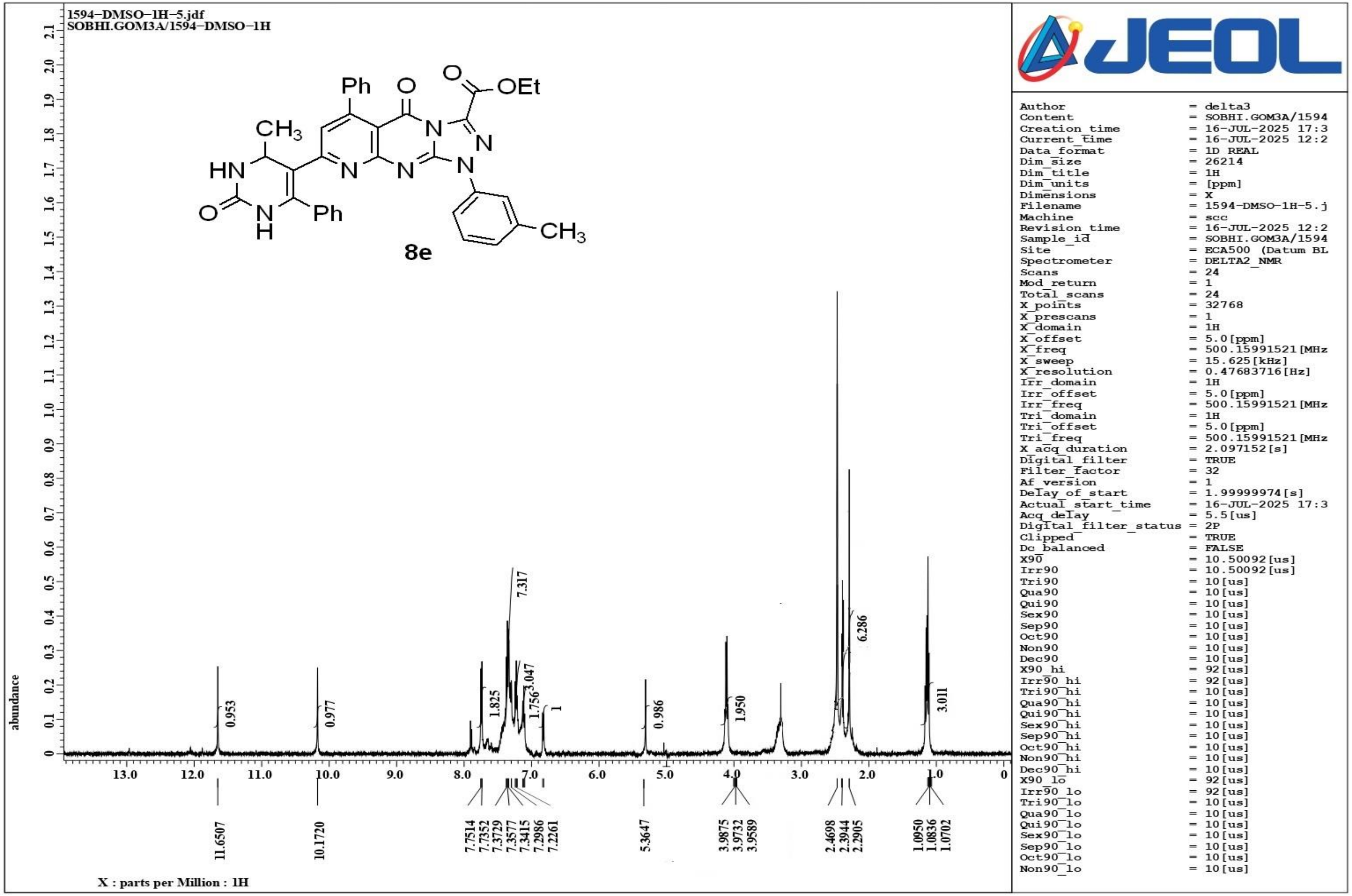

^{1}H-NMR spectra of compound **8e**

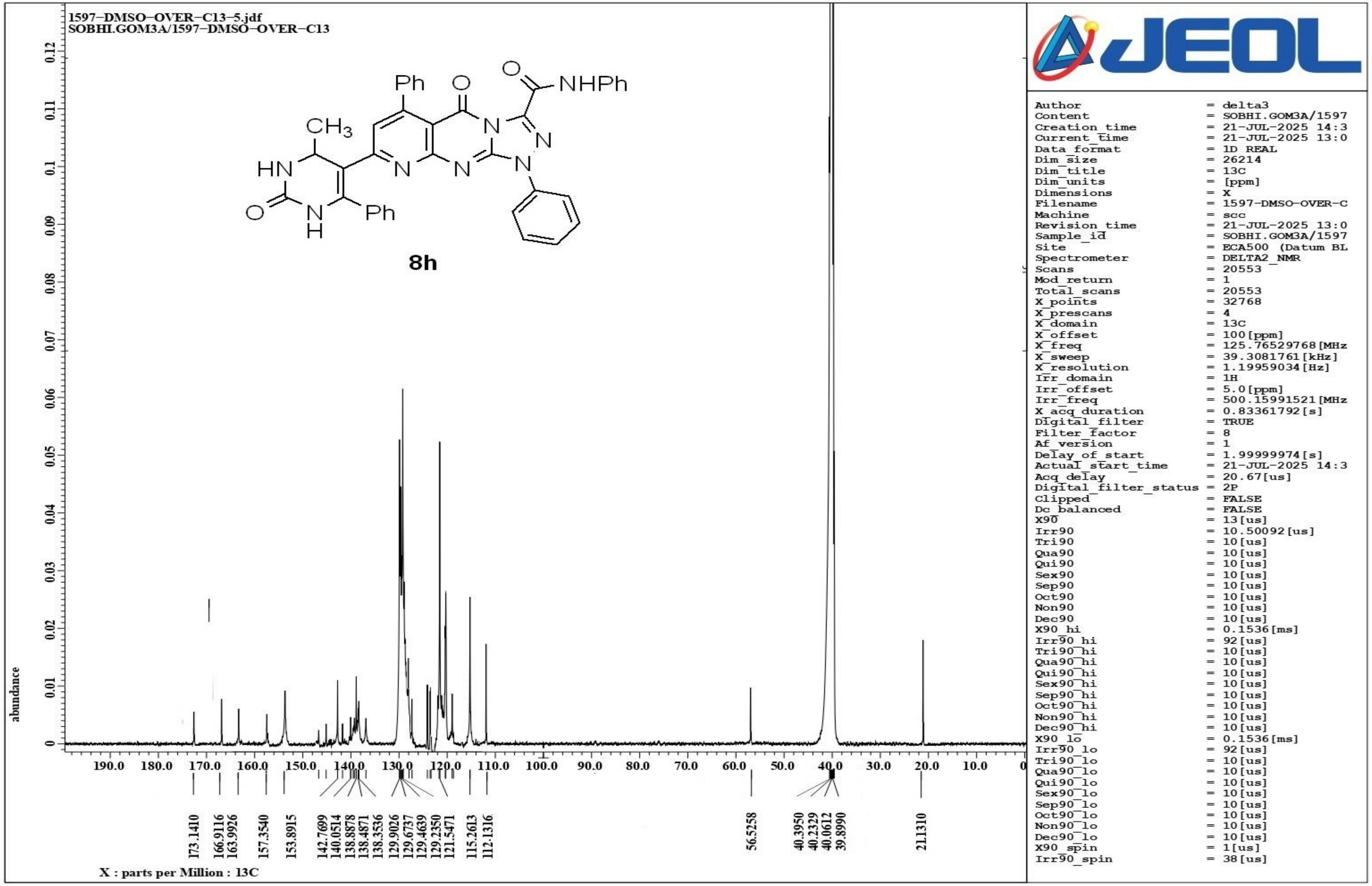

^{13}C-NMR spectra of compound **8h**

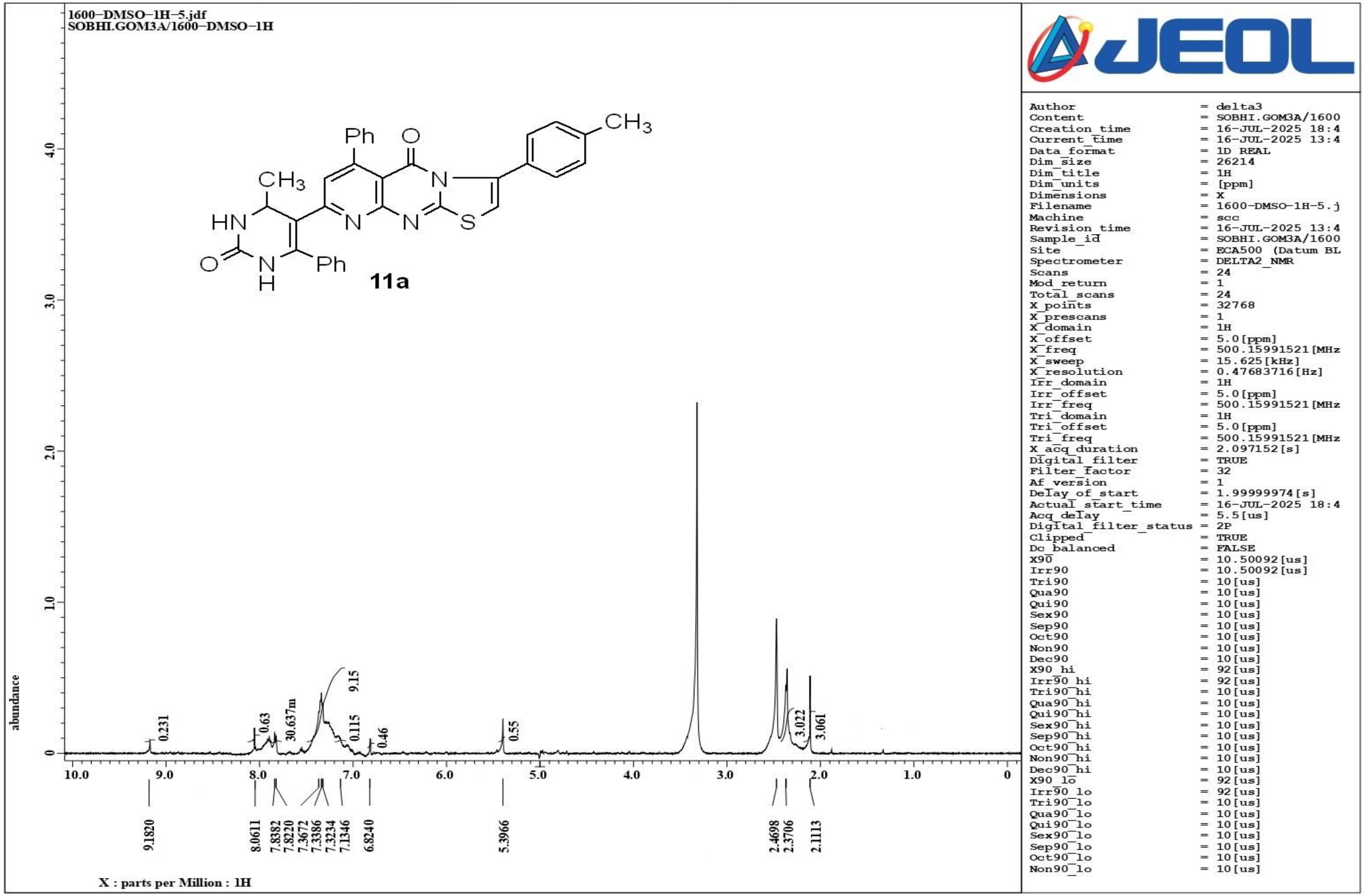

^{1}H-NMR spectra of compound **11a**

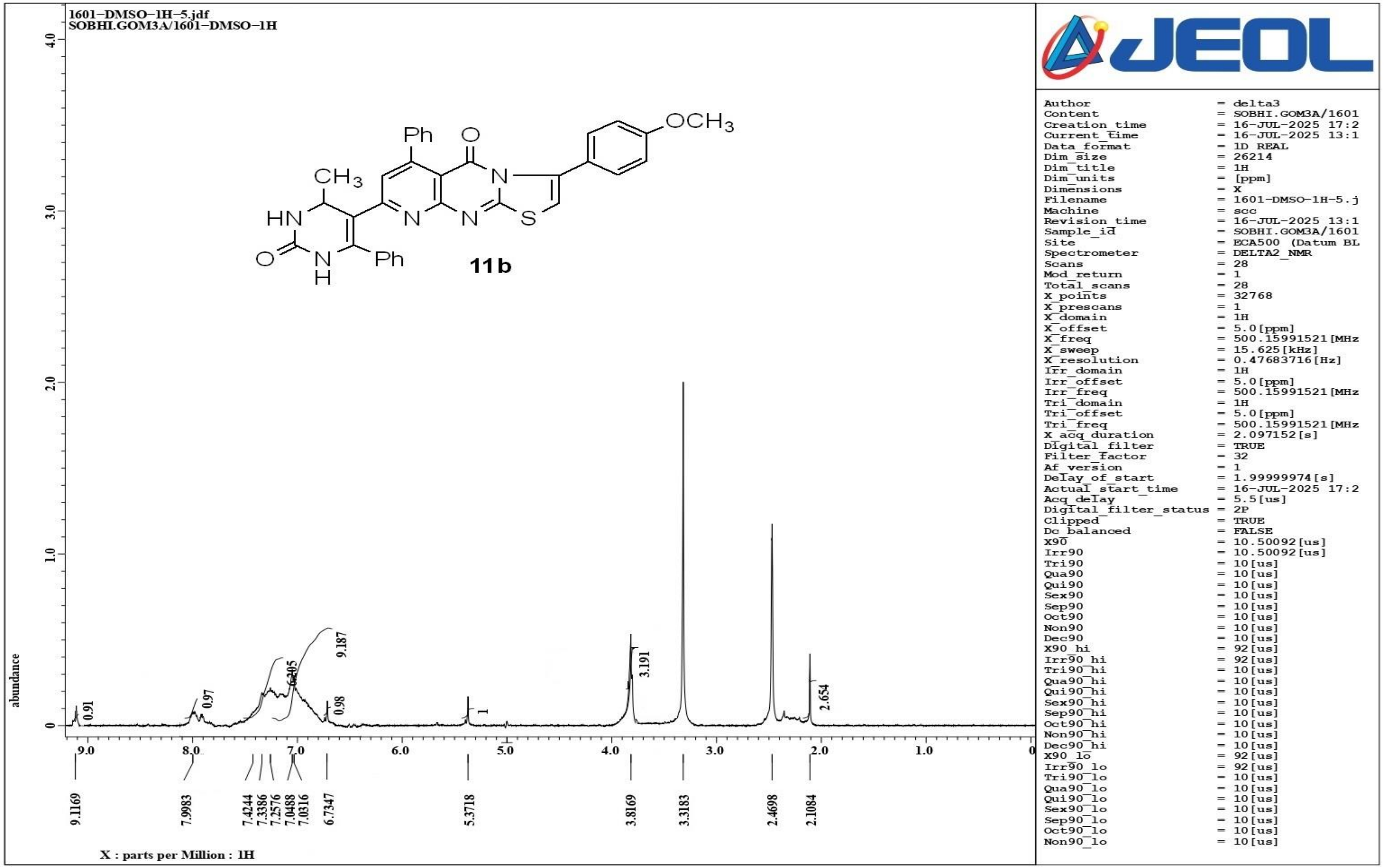

^{1}H-NMR spectra of compound **11b**

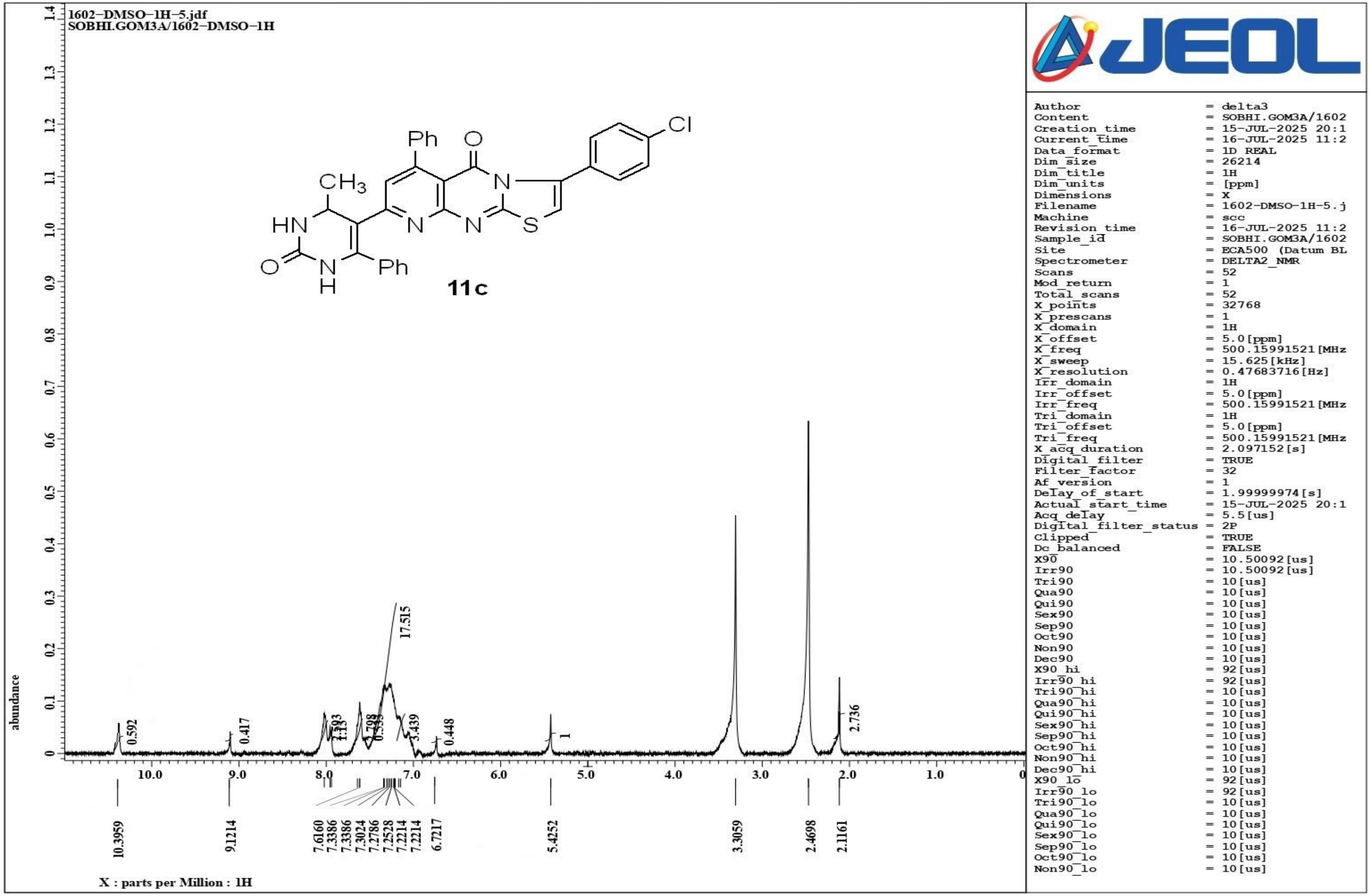

^{1}H-NMR spectra of compound **11c**
